# The History of Coast Salish ‘Woolly Dogs’ Revealed by Ancient Genomics and Indigenous Knowledge

**DOI:** 10.1126/science.adi6549

**Published:** 2023-12-14

**Authors:** Audrey T. Lin, Liz Hammond-Kaarremaa, Hsiao-Lei Liu, Chris Stantis, Iain McKechnie, Michael Pavel, Susan sa'hLa mitSa Pavel, Senaqwila Sen̓áḵw Wyss, Debra qwasen Sparrow, Karen Carr, Sabhrina Gita Aninta, Angela Perri, Jonathan Hartt, Anders Bergström, Alberto Carmagnini, Sophy Charlton, Love Dalén, Tatiana R. Feuerborn, Christine A.M. France, Shyam Gopalakrishnan, Vaughan Grimes, Alex Harris, Gwénaëlle Kavich, Benjamin N. Sacks, Mikkel-Holger S. Sinding, Pontus Skoglund, David W.G. Stanton, Elaine A. Ostrander, Greger Larson, Chelsey G. Armstrong, Laurent A.F. Frantz, Melissa T.R. Hawkins, Logan Kistler

**Affiliations:** 1 Department of Anthropology, National Museum of Natural History, Smithsonian Institution, Washington DC, USA; 2 Vancouver Island University, Nanaimo, BC, Canada; 3 Department of Geology and Geophysics, University of Utah, Salt Lake City, UT, USA; 4 Department of Anthropology, University of Victoria, Victoria, BC, Canada; 5 Twana/Skokomish Indian Tribe. Skokomish Nation, WA, USA; 6 Coast Salish Wool Weaving Center, Skokomish Nation, WA, USA; 7 Skwxwú7mesh Úxwumixw (Squamish Nation), North Vancouver, BC, Canada; 8 Musqueam First Nation, Vancouver, BC, Canada; 9 Karen Carr Studio, Silver City, NM, USA; 10 School of Biological and Behavioural Sciences, Queen Mary University of London, London, UK; 11 Department of Anthropology, Texas A&M University, College Station, TX, USA; 12 Chronicle Heritage, AZ, USA; 13 Indigenous Studies, Simon Fraser University, Burnaby, BC, Canada; 14 Ancient Genomics Laboratory, The Francis Crick Institute, London, UK; 15 School of Biological Sciences, University of East Anglia, Norwich, UK; 16 Palaeogenomics Group, Department of Veterinary Sciences, Ludwig Maximilian University Munich, Munich, Germany; 17 PalaeoBARN, School of Archaeology, University of Oxford, Oxford, UK; 18 BioArCh, Department of Archaeology, University of York, York, UK; 19 Centre for Palaeogenetics, Stockholm, Sweden; 20 Department of Zoology, Stockholm University, Stockholm, Sweden; 21 Center for Evolutionary Hologenomics, The Globe Institute, University of Copenhagen, Copenhagen, Denmark; 22 National Genome Research Institutes, National Institutes of Health, Bethesda, MD, USA; 23 Museum Conservation Institute, Smithsonian Institution, Suitland, MD, USA; 24 Memorial University of Newfoundland, St. Johns, NL, Canada; 25 Mammalian Ecology and Conservation Unit, Veterinary Genetics Laboratory, School of Veterinary Medicine, University of California Davis, Davis, CA, USA; 26 Department of Population Health and Reproduction, School of Veterinary Medicine, University of California-Davis, Davis, CA, USA; 27 Department of Biology, University of Copenhagen, Copenhagen, Denmark; 28 Cardiff School of Biosciences, Cardiff University, Cardiff, UK; 29 Department of Vertebrate Zoology, National Museum of Natural History, Smithsonian Institution, Washington DC, USA

## Abstract

Ancestral Coast Salish societies in the Pacific Northwest kept long-haired “woolly” dogs that were bred and cared for over millennia. However, the dog wool-weaving tradition declined during the 19^th^ century, and the population was lost. Here, we analyze genomic and isotopic data from a preserved woolly dog pelt, “Mutton”, collected in 1859. Mutton is the only known example of an Indigenous North American dog with dominant pre-colonial ancestry postdating the onset of settler colonialism. We identify candidate genetic variants potentially linked with their unique woolly phenotype. We integrate these data with interviews from Coast Salish Elders, Knowledge Keepers, and weavers about shared traditional knowledge and memories surrounding woolly dogs, their importance within Coast Salish societies, and how colonial policies led directly to their disappearance.

Dogs were introduced to the Americas from Eurasia via northwestern North America ~15,000 years ago, and have been ubiquitous in Indigenous societies of the Pacific Northwest (PNW) for millennia (1–4). Coast Salish peoples in the Salish Sea region (Fig. 1A) kept multiple different types of dogs: hunting dogs, village dogs, and “woolly dogs” with a thick woolen undercoat that was shorn for weaving (4, 5). Dog wool blankets, often blended with mountain goat wool, waterfowl down, and plant fibers like fireweed and cattail fluff, were prestigious cultural belongings (6–8). Woolly dogs, known as sqwemá:y, ske’-ha, sq^w^ǝméy̓, sq^w^baý, and **QebeO** in some Coast Salish languages (9), were emblems of some communities, as depicted in a 19^th^ century Skokomish/Twana basket (Fig. 1B (10)).

The first comprehensive book on Salish weaving (11) scrutinized most Coast Salish woven blankets in museums around the world, questioning if any contained primarily dog wool, and disputing the fiber’s spinnability. More recent proteomic analysis of 19^th^ century blankets confirmed the use of dog wool in Coast Salish weaving (12). In addition, zooarchaeological remains thought to be from woolly dogs have been found in dozens of archaeological sites in Coast Salish territories beginning ~5,000 years before present (BP) (2, 4) (Fig. 1A). The last Coast Salish woolly dogs likely lived in the late 19th/early 20th centuries (5, 13). Later photographs and records referring to woolly dogs extend into the 20^th^ century, but these examples likely reflect mixed ancestry or non-Indigenous breeds (9).

The decline in dog wool weaving has previously been attributed to the proliferation of machine-made blankets by British and American trading companies in the early 19^th^ century (11, 13). However, this explanation ignores the cultural importance of woolly dogs, as reflected through their enduring use by weavers, particularly for high status items like regalia (7, 14). Given their role in Coast Salish societies, it is unlikely that the entire dog wool tradition would have been abandoned simply because of the ready availability of imported textiles. Further, this explanation ignores weavers’ efforts to maintain culturally relevant practices in the face of settler colonialism. The use of blankets and robes served not only a functional purpose, but also a spiritually protective role in Coast Salish cultures. Wearing a ceremonial blanket was spiritually transformative since it intertwined the creator of the blanket, the wearer, and the community (13–15).

The only known pelt of an extinct Coast Salish woolly dog is of “Mutton”, a dog cared for by naturalist and ethnographer George Gibbs during the Northwest Boundary Survey (1857-1862). According to Gibbs’s field journal and Smithsonian ledgers (USNM A4401-A4425), Mutton became ill and died in late 1859 (9, 15). His pelt and lower leg bones are housed at the Smithsonian Institution (USNM 4762) (Figs. S2, S4).

Here, we combine genomic analysis, ethnographic research, stable isotope and zooarchaeological analysis, and archival records to investigate this iconic dog’s history, including ancestry, the genetic underpinnings of woolliness, and their ultimate decline. We sequenced Mutton’s nuclear genome to a mean 3.4x depth of coverage and, for comparison, a non-woolly village dog (Figs. S3, S5) from the nearby Semiahmoo Bay region to low coverage (0.05x; “SB dog” hereafter, USNM 3512; collected 1858). For additional genomic context, we increased the coverage of an ancient dog from Port au Choix, Newfoundland (AL3194; 4,020 cal BP) (3), from 1.9x to 11.9x, and sequenced the genome of an ancient dog from Teshekpuk Lake, Alaska (ALAS_015; 3,763 BP; 1.23x), three modern coyotes, and 59 modern dogs representing 21 breeds (DataS1). We also undertook δ^13^C and δ^15^N stable isotope analysis of Mutton and the SB dog to test for substantial differences in their dietary life histories. Finally, we interviewed seven Coast Salish Elders, Knowledge Keepers, and wool weavers about family histories and traditional knowledge surrounding woolly dogs to provide a cultural framework for interpreting the genomic analyses (9). The interviewees span several Coast Salish communities, including Stó:lō, Squamish, Snuneymuxw, and Musqueam Nations in British Columbia (BC) and Suquamish, and Skokomish/Twana in Washington.

## Woolly dog origins

Throughout northwestern North America there are numerous oral histories and origin stories involving the woolly dog. Skokomish/Twana Elder, Michael Pavel, reports that in a former time, when all beings including woolly dogs were recognized as relatives, all were ‘people’ and were family. High-status Qw’ó:ntl’an women are an example of those who trace their lineages from the woolly dog at a time when all beings were one family (16). According to Pavel: “*…And out of [the origin story], [woolly dogs] were given the gift of the wool, and they were able to teach the women how to gather the wool, how to process the wool, how to spin the wool, and how to weave with the wool*” (9).

Early colonial explorers and scholars speculated that woolly dogs originated in Japan (17) or were recently introduced to the Coast Salish by Dene from their homelands in northern boreal Canada (18). However, zooarchaeological remains of morphologically distinct dogs in Coast Salish territories suggest woolly dog husbandry was present for ~5,000 years before European colonization (2, 4). Furthermore, longstanding oral histories and traditional knowledge hold that woolly dogs have been part of Coast Salish society for millennia (9).

**Figure 2:**
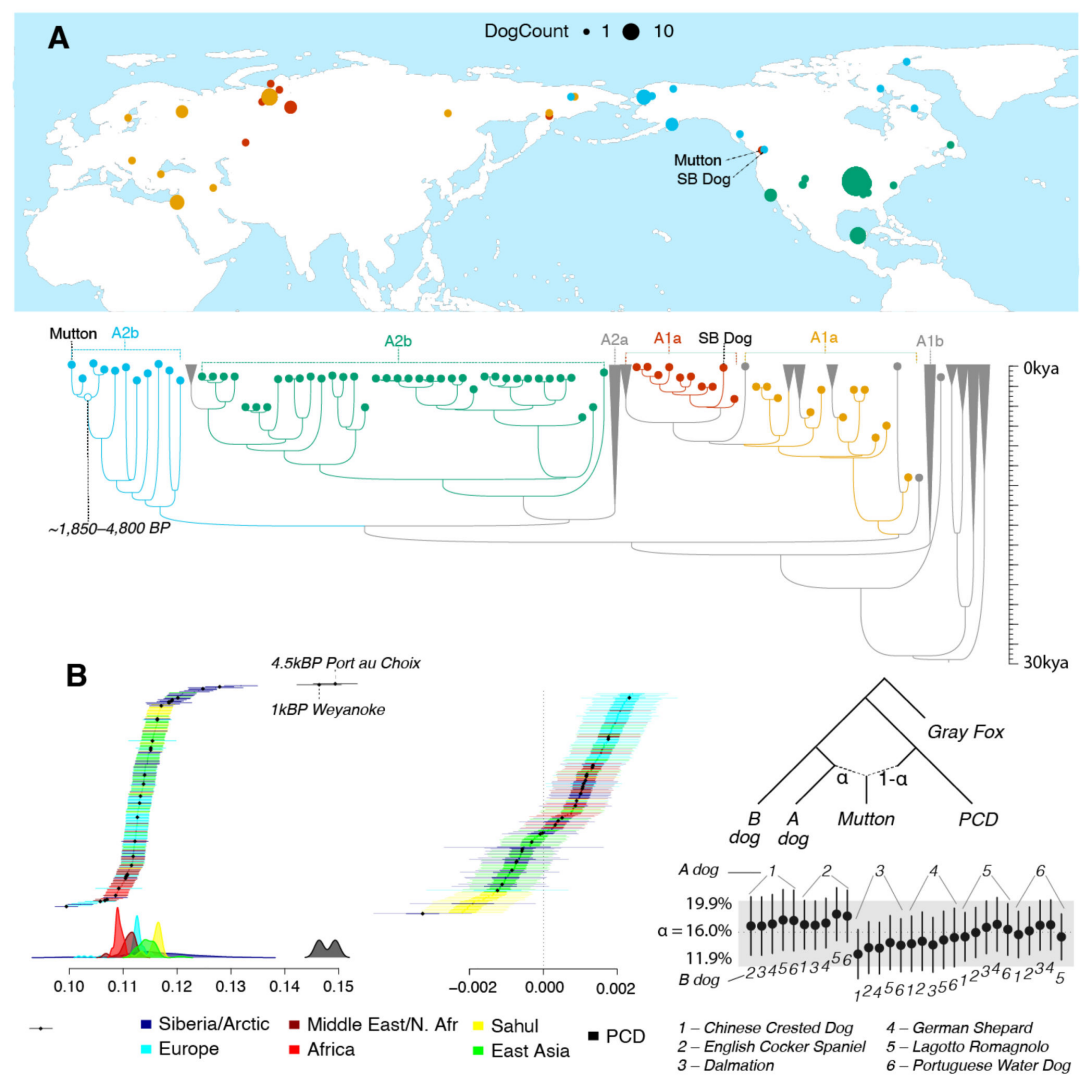
Genetic ancestry of woolly dogs. **2A**. mtDNA tree of 207 dogs with A2b (Mutton) and A1a (SB Dog) haplotypes expanded. Map points correspond to colored tree tips for the most similar archaeological and historic dog mtDNAs, highlighting the subclades of interest and the broader haplotypes. Samples used are listed in DataS1. **2B**. Outgroup*-f3* statistics (*f*3(GrayFox; Mutton, B) or estimation of shared drift between Mutton and 229 other dogs reveals that Mutton has highest similarity to PCDs. Black point estimates indicate ancient genomes. **2C**. D-statistics (((PCD, Mutton), Test Dog), Gray Fox) consistent with gene flow into Mutton’s background, with European breeds appearing the most likely contributors to Mutton’s non-PCD ancestry. **2D**. *f4*-ratio tests (*f4*(A, Out; Mutton, AL3194-PortauChoix): *f4*(A, Out; B, AL3194-PortauChoix)) to estimate the proportion of European settler dog ancestry in Mutton’s background using six modern European breeds as proxies for Mutton’s European ancestry component.

To test whether Mutton has pre-colonial or settler dog ancestry, we first compared his mitochondrial genome to 207 ancient and modern dogs from a global sampling. Mutton carries the A2b mtDNA haplotype, which emerged after dogs initially arrived from Eurasia (3). Most of this mtDNA lineage of so-called pre-colonial dogs (PCDs) disappeared after European colonization (3, 19, 20). Mutton’s nearest mtDNA neighbor is an ancient dog (PRD10, ~1,500 BP) from Prince Rupert Harbour, BC (Figs. 2A, S16). PRD10 is the only archaeological dog from the PNW in the mtDNA dataset, and this similarity reflects the deep roots of Mutton’s maternal ancestry in the region. A pair of modern and ancient (~620 BP) dogs from Alaska form a sister clade of the Mutton-PRD10 grouping, further underscoring the long-term maternal population structure in northwestern North America. In contrast, the SB dog carries an A1a haplotype, similar to most modern European dogs, and the most common present-day haplotype worldwide (64 out of 207 dogs in our analysis) (21).

To place a timeframe on the divergence of Mutton’s maternal lineage, we performed a molecular clock analysis on the mitochondrial phylogeny (DataS1). The results suggest a mitochondrial common ancestor estimated between 4,776 and 1,853 years BP for the subclade containing Mutton, PRD10, and the two Alaskan dogs (95% highest posterior density; Figs. 2A, S16). Although we are limited by the analysis of a single individual, this timing is generally consistent with the increasing occurrence of small sized ‘woolly’ dog zooarchaeological remains in the regions surrounding the Salish Sea (2).

To assess Mutton’s nuclear ancestry, we analyzed 217 globally distributed ancient and modern dogs. Outgroup*-f3* statistics reveal that Mutton carries substantially greater shared genetic drift with PCDs than with any other dogs, specifically, archaeological remains of a dog from Port au Choix, Newfoundland (4,020 cal BP), and from Weyanoke Old Town, Virginia (~1,000 BP) (Figs. 2B, S17). Since Mutton lived after European colonization and waves of pre-colonial dog introductions (3, 21), we tested for gene flow from introduced lineages using D-statistics. We found that European breeds yielded strongly positive D-statistics, indicating that Mutton’s non-PCD ancestry most likely stemmed from introduced European dogs (Fig. 2C).

To refine these results, we used *f4*-ratio tests with six modern European breeds (Chinese Crested dog, English Cocker Spaniel, Dalmatian, German Shepherd, Lagotto Romagnolo, and Portuguese Water Dog), estimating that Mutton had 84% PCD and 16% European ancestry (11.9%–19.9% 2 SE range; Fig. 2D). The *f4*-ratio test may slightly over-estimate Mutton’s European ancestry if the true contributor of this ancestry was equally related (an outgroup) to the two European breeds in the tests. However, estimates across all permutations are broadly consistent (Figs. 2D, S18), suggesting European ancestry roughly on the order of one great-grandparent in Mutton’s background. In contrast, outgroup*-f3* statistics indicate that the contemporaneous SB dog appears highly admixed, showing greatest similarity to ancient dogs from Siberia and Alaska (Fig. S17). The distribution of PCD vs. European ancestry tracts in Mutton can provide some additional insight into the timing of admixture. Although this method is imprecise due to recent admixture and the scarcity of PCD source population data, we estimate that Mutton’s European admixture occurred 10.8±4.9 generations before (1 SE). Assuming a three-year generation time, this analysis suggests admixture ~32 years before Mutton’s birth, consistent with post-colonial admixture (9).

To test for dietary differences between Mutton and the SB dog, we performed stable isotope analysis of δ^13^C and δ^15^N on bone collagen and hair keratin. The SB dog has high δ^13^C and δ^15^N values similar to archaeological dogs from the PNW (22), indicating a traditional marine-based diet (Figs. S13-S14). Mutton’s isotope values reveal a more terrestrial and C3-rich diet, likely reflecting Mutton’s life and travels with Gibbs from an early age (Figs. S14-B,C, S15, (9)).

The persistence of a high proportion of post-colonial PCD ancestry may reflect concerted efforts by Coast Salish peoples to maintain the breed against the pressure of gene flow from non-native dogs. Mutton lived near the end of traditional woolly dog husbandry (5, 9, 13). Although he had mixed ancestry, Mutton’s background is dominated by PCD ancestors, compared to the contemporaneous SB dog. This may indicate careful reproductive management to maintain woolly dogs’ unique genetic makeup and phenotype until their decline. Mutton’s fraction of European ancestry also highlights the turbulent cultural moment when Mutton lived and illustrates how interbreeding with settler-introduced dogs could have threatened the survival of woolly dogs.

## The influence of people on the woolly dog genome

Woolly dogs were treated as beloved extended family members. According to Debra qwasen Sparrow, a Musqueam Master weaver, her grandfather [Ed Sparrow, (1898-1998)] told her “*every village had [woolly dogs], that they were like gold because they were mixed with the mountain goat and then rove and spun*” (9). Dogs also comprised a form of wealth and status for Coast Salish women, who carefully managed the dogs to maintain their woolly coats, isolating them on islands or in pens to strictly manage their breeding (9, 17, 23). Often island names reflect their connection with dogs, such as *sqwiqwmi*’ (“Little Dog”) village on Cameron Island in Nanaimo, Snuneymuxw territory, British Columbia. The prevention of interbreeding wool dogs with hunting or village dogs was critical for maintaining their unique hair characteristics: soft guard hairs with an unusually long crimpy undercoat (Fig. S2), which was highly spinnable and made warm blanket yarn. These management practices likely contributed to Mutton’s PCD ancestry long after the onset of settler colonialism.

**Figure 3:**
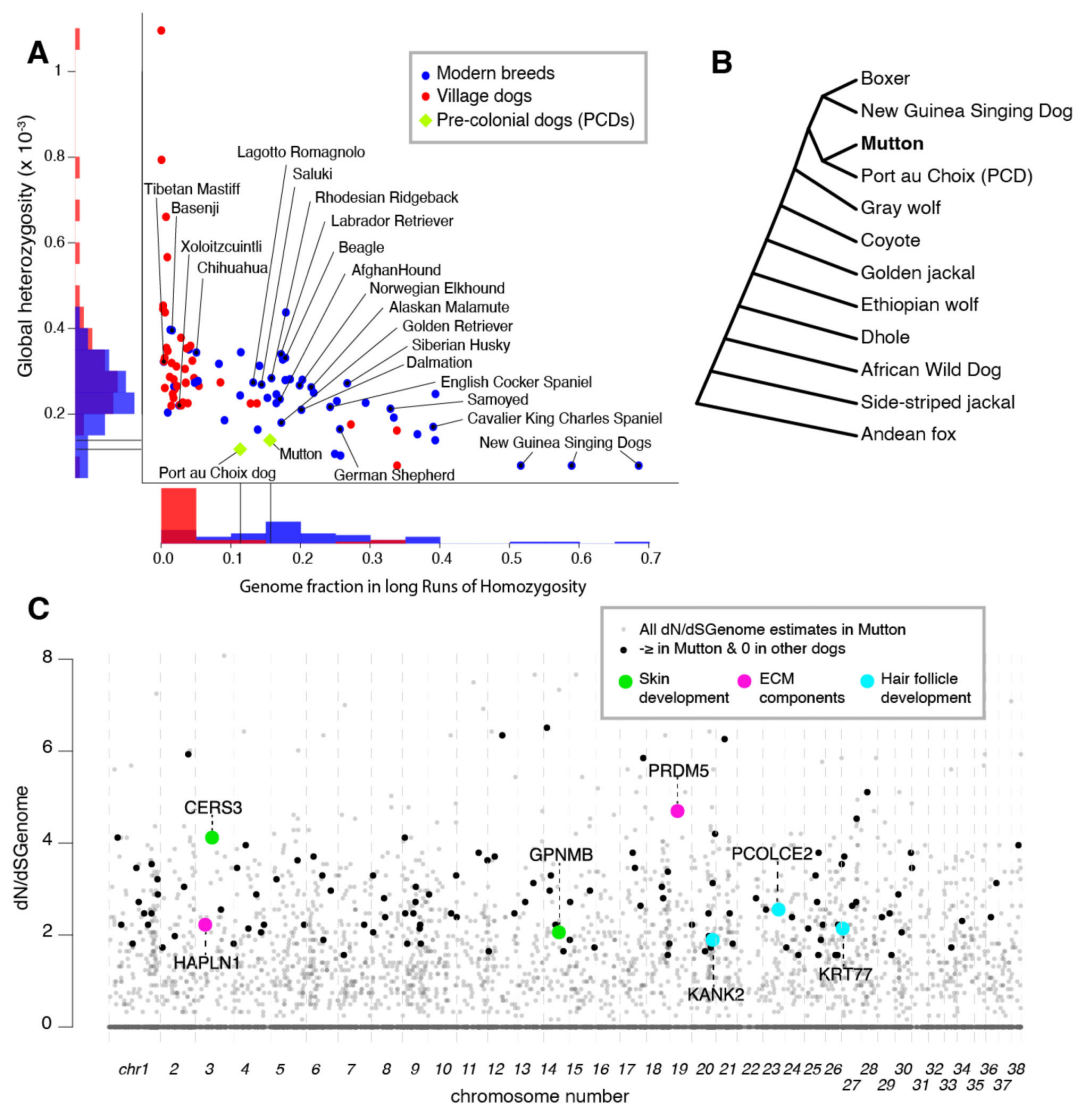
Genomic outcomes of management and selection. **3A**. Global heterozygosity and long runs of homozygosity over transversions in Mutton compared to modern dogs and the ancient Port au Choix dog. All dogs have been downsampled to Mutton’s coverage level for analysis. **3B**. Tree schematic used in dN/dS analysis to identify genes under selection in Mutton compared to other canids. Branching order after (50). dN/dS estimates were done separately including one of the four dogs plus all other canids. Genes with elevated dN/dS_Genome_ values in multiple dogs could reflect more ancient shared selection before the separation of the woolly dog lineage. Therefore, likely candidates for selection in woolly dogs were conservatively assessed where dN/dS_Genome_>1.5 in Mutton (9), but dN = 0 in the other three dogs, including one PCD. **3C**. Genes with an excess of non-synonymous mutations in Mutton. Black points are the 125 selection candidates on the basis of dN/dS_genome_ ≥1.5 in Mutton but dN=0 in three other dogs including one PCD (9). Several genes with high dN/dS_genome_ in Mutton (shown in gray) are excluded as selection candidates because they carry at least one non-synonymous mutation in other dogs. This approach is designed to conservatively highlight genes where selection is more likely specific to Mutton’s lineage rather than during dog domestication or in the common ancestors of PCDs. Candidate genes discussed in text are indicated.

Long-term husbandry for woolly hair likely limited woolly dogs’ effective population size, which would be reflected in nucleotide diversity and thus in Mutton’s heterozygosity. We found that Mutton’s heterozygosity is in the lowest range of living breeds (n=51) and village dogs (n=42) downsampled to the same coverage (Fig. 3A). Additionally, runs of homozygosity (ROH) better reflect recent demography than global heterozygosity. Using an ROH method optimized for low coverage (9, 24), we estimate that 15.7% of Mutton’s genome is in ROH of 2.5Mbp or greater, again in the range of modern breeds. The ancient Port au Choix dog also has low genomic heterozygosity and 11.3% ROH, so Mutton’s low heterozygosity may partly reflect shared demographic history from a small PCD founding population (Fig. 3A). Because of recent European admixture, Mutton’s genome is inevitably more heterozygous than his recent woolly dog ancestors.

To search for evidence of genetic mechanisms for woolliness, we used maximum likelihood-based estimation of the enrichment of non-synonymous mutations (dN/dS) observed within Mutton’s coding regions (9). We evaluated 11,112 genes with sufficient sequence coverage for all dogs and outgroups (DataS1), and restricted selection candidate identification to genes with elevated dN/dS in Mutton but lacking any non-synonymous mutations in three other dogs, including one PCD (Fig. 3B). Although power to detect selection is fundamentally limited with only a single genome, we identified a candidate set of genes with high lineage-specific dN/dS values. We identified 125 genes as candidates for positive selection in woolly dogs (DataS2). Among these, 28 have plausible links to hair growth and follicle regeneration based on a model of the hair growth cycle (Fig. S12), and are associated with cell replication, proliferation, the formation of extracellular matrix components, vascularization, and related processes (25–31) (Fig. 3C, DataS3).

Candidate selection genes in Mutton include *KANK2*, a steroid signaling regulator responsible for hereditary diseases of the hair shaft in humans (32). A unique non-synonymous mutation in Mutton lies in the adjacent amino acid to the *KANK2* mutation causing a “woolly” hair phenotype in humans (32). *KRT77* is a member of the keratin gene family responsible for the structural integrity of cells in the epithelium and hair follicles. Mutations in keratin genes are linked to curly hair phenotype in other dogs, rats, and mice (31), woolly hair and hereditary hair loss in humans (26, 30), and multiple *KRT* genes underwent selection in woolly mammoths (25). *CERS3, PRDM5, HAPLN1* are associated with maintaining the integrity of the skin or connective tissue in humans (27, 28). *GPNMB* is involved in multiple cellular functions in the epidermis, potentially mediating pigmentation (29). We also manually evaluated 15 specific variants from previous literature linked with hair characteristics in living dog breeds (DataS4). Apart from a widespread *FGF5* mutation conferring long hair (33, 34), Mutton showed the ancestral allele in all cases with data present (DataS4), illustrating the independent origins of woolly dogs’ unique phenotype.

## The impact of colonialism on the iconic breed’s disappearance

Woolly dogs’ decline throughout the 19^th^ century is not fully understood. The narrative that the influx of trade blankets into the region led to the abandonment of woolly dog husbandry oversimplifies a complex scenario. By 1857 (a year before Mutton’s birth) in Sto:lo territory, where Mutton was most likely acquired, the settler population consisted of only a few dozen permanent settlers at Fort Langley (35, 36). The following year, more than 33,000 miners arrived at present-day British Columbia during the 1858 Fraser River Gold Rush. This large-scale migration set off conflicts between miners, colonial governments, and Indigenous peoples. Meanwhile, Indigenous populations declined by an estimated two-thirds between 1830 and 1882 (37). Smallpox epidemics—almost one every generation from the 1700s to 1862 (38)—are estimated to have killed more than 90% of Indigenous people in some villages across BC (38), along with steady depopulation due to other introduced diseases such as mumps, tuberculosis, and influenza (37).

Survival of woolly dogs depended upon the survival of their caretakers. In addition to disease, expanding colonialism increased cultural upheaval, displacement of Indigenous peoples, and a diminished capacity to manage the breed. Policies targeted Indigenous governance and inherent rights, resulting in the deliberate disenfranchisement and criminalization of Indigenous cultural practices (39). Indigenous women, the caretakers of woolly dogs and weaving knowledge, were specifically targeted. Missionization efforts reduced women’s roles in society, and legislation such as the Indian Act (1876) explicitly prohibited women from participating in local governance, denied women basic property rights, and restricted their movement (39). In the 20^th^ century, transference of cultural knowledge was further disrupted by mandatory residential schooling designed to remove children from their families and suppress culture (40).

Through these compounding waves of colonialism, the transmission of important knowledge relating to the husbandry of the woolly dog, processing the hair, spinning, and weaving was interrupted. Stó:lō Elder Rena Point Bolton, 95 years old in 2022, recalls how Th’etsimiya, her great-grandmother, had kept woolly dogs, but was forced to give them up: *“They were told they couldn’t do their cultural things. There was the police, the Indian Agent and the priests. The dogs were not allowed. She had to get rid of the dogs.”* (9). The dogs represented high status and traditional practices that threatened British and later Canadian dominion, and as such were removed via policies of assimilation (40–42). The weaving traditions were not completely lost, as many cultural teachings and types of expertise were carried on in secret. Bolton said: “*Our people were not allowed to spin on shxwqáqelets [traditional spindle whorls]. They could spin on a European one but not on the shxwqáqelets. They couldn’t use their looms, and they would take them out and burn them or they would give them to museums or collectors…The generation that was there when the Europeans came and colonized us, that’s where it ended, and there [were] just a few people who went underground. And my grandmother and my mother were two of them*.” (9).

A growing body of research demonstrates how peoples of the PNW cared for and managed their ancestral lands, cultivating diverse and highly localized plants and marine foods (43–45). Woolly dogs may have also been similarly localized and diverse. We focus on Coast Salish dogs, but non-Salish peoples in the PNW also kept woolly dogs. For example, Nuu-chah-nulth peoples of western Vancouver Island kept a different wool dog that were reportedly bigger and had coats of different colors including brown, spotted, black, grey, or white (46–48). These differences could be population-specific, or they could be a result of widespread phenotypic diversity, as noted by explorers in the 18^th^ and 19^th^ centuries (17), reflecting trade among the different Indigenous communities.

Weaving and woolly dogs are intertwined in Coast Salish culture and society, which cannot be separated from the long-time management of their ancestral homelands. Weavers, artists, and Elders continue to promote the renewal of traditional or customary weaving knowledge and practices. Artist Eliot Kwulasultun White-Hill (Snuneymuxw) said (9): *“It starts to unravel, in a way, people’s understanding of us as a hunter gatherer society… Our relationship with the woolly dogs, our relationship with the camas patches and the clam beds, the way that we tended the land and tended the forests… these all show the systems in place that are far more complex than what people take for granted about Coast Salish culture.”*

## Supplementary Material

DataS1

DataS2

DataS3

DataS4

DataS5

Supplementary Materials

## Figures and Tables

**Figure 1:**
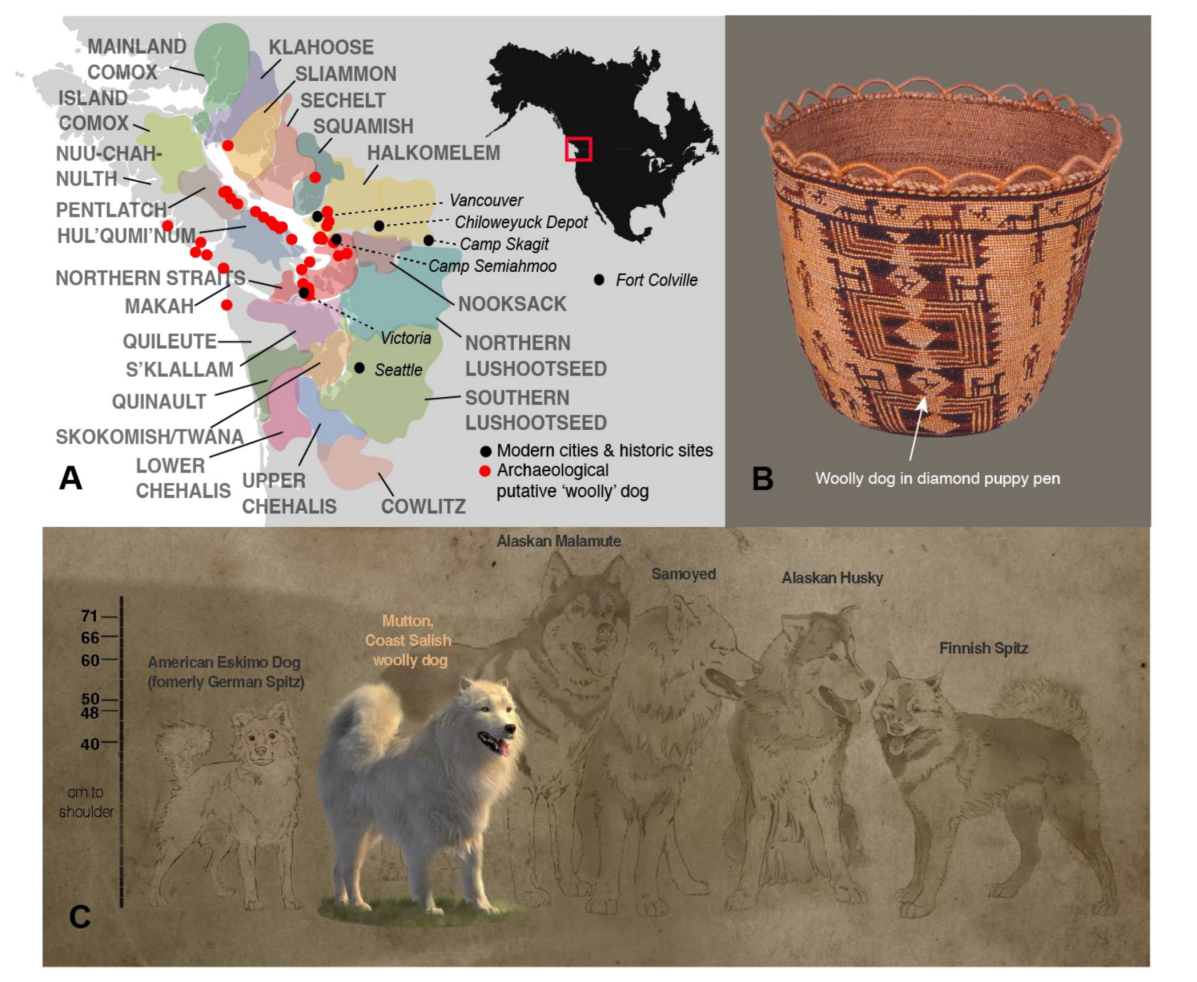
Domestic dogs in the culture and society of Indigenous Coast Salish peoples. **1A**. Coast Salish ancestral lands include the inner coastal waterways of Salish Sea in southwest British Columbia and Washington State. Archaeological woolly dog data are from (2). Distribution of the Coast Salish languages in the 19th century as indicated by colored areas. The map is modified from https://commons.wikimedia.org/wiki/File:Coast_Salish_language_map.svg and licensed under CC BY-SA 4.0. **1B**. Woven Skokomish/Twana basket with woolly dog iconography, depicted with upturned tails. Woolly dog puppies are inside pens represented by diamond shapes (10) (courtesy of Burke Museum, Catalog number #1-507). **1C**. Forensic reconstruction of a woolly dog based on Mutton’s pelt measurements and archaeological remains (9). Sketches of Arctic and spitz dog breeds are shown for scale and comparison of appearance, and do not imply a genetic relationship.

## Data Availability

Genomic sequencing data for Mutton, SB dog, the Port au Choix dog (AL3194), and ALAS_015 are available for non-commercial use via NCBI SRA Project Accession PRJNA1005336 and BioSample Accessions SAMN36985984-SAMN36985987. The SRA Project Accession for the modern coyote from Wyoming is PRJNA734649. Stable isotope data are available (49). All other public genomic data sources are provided in DataS1.

## 1 sentence summary

A 19th century dog genome and Traditional Knowledge illuminate the life, history, and decline of Coast Salish woolly dogs
