# Supplementary material for: The History of Coast Salish ‘Woolly Dogs’ Revealed by Ancient Genomics and Indigenous Knowledge
Source: Science. Author manuscript; Available in PMC 2024 Jan 29. (PMC7615573; doi:10.1126/science.adi6549)
Supplement: Supplementary Materials [file EMS193513-supplement-Supplementary_Materials.pdf]

Science
AAAS

Supplementary Materials for

The History of Coast Salish 'Woolly Dogs' Revealed by Ancient Genomics and Indigenous Knowledge

Audrey T. Lin[1]*, Liz Hammond-Kaarremaa[1,2]*, Hsiao-Lei Liu[1], Chris Stantis[1,3], Iain McKechnie[4], Michael Pavel[5], Susan sa'hLa mitSa Pavel[5,6], Senaqwila Seṅáḵw Wyss[7], Debra qwasen Sparrow[8], Karen Carr[9], Sabhrina Gita Aninta[10], Angela Perri[11,12], Jonathan Hartt[13], Anders Bergström[14.15], Alberto Carmagnini[16], Sophy Charlton[17.18], Love Dalén[19,20], Tatiana R. Feuerborn[21,22], Christine A.M. France[23], Shyam Gopalakrishnan[21], Vaughan Grimes[24], Alex Harris[22], Gwénaëlle Kavich[23], Benjamin N. Sacks[25,26], Mikkel-Holger S. Sinding[27], Pontus Skoglund[14], David W.G. Stanton[16,28], Elaine A. Ostrander[22], Greger Larson[17] Chelsey G. Armstrong[13], Laurent A.F. Frantz[10,16], Melissa T.R. Hawkins[29], Logan Kistler[1]*

*Corresponding author. Email: linat@si.edu (A.T.L.); liz.hammond-kaarremaa@viu.ca (L.H.-K.); kistlerl@si.edu (L.K.)

The PDF file includes:

Materials and Methods
Figs. S1 to S19
Tables 1 and 2
References

Other Supplementary Materials for this manuscript include the following:

DataS1 to S5

I. Materials

Archaeological/historic samples and context

Wool dogs in some Coast Salish languages
Halq’emelem: sqwemá:y (*51*)
Hul’q’umi’num’: sqwumey'
Lushootseed: sqí aʔ or ske'-ha (*52*)
Lower Cowlitz: kimia (*53*)
Samish: sqʷəméý̓ (*54*)
SENĆOŦEN (Saanich): sqʷəméý̓ (*54*)
Tuwaduq: QebeO or qaQebeO (*55*)
Twana: Sqwbaý (*13*)

Documentary evidence of purported woolly dogs
The most famous contemporary depiction of the Coast Salish weaving complex is a painting by Paul Kane, “A Woman Weaving a Blanket” 912.1.93, **Fig. S1**), painted two years after Kane visited the PNW and did a few sketches while visiting Southern Vancouver Island in 1847. His original sketch of the dog is more detailed than the dog featured in the painting. Kane had observed, "They have a peculiar breed of small dogs with long hair of a brownish black and a clear white” (*56*).

There are several well-known 20th century photographs referring to purported woolly dogs. A photograph dated 1912, taken by anthropologist John Douglas Leechman (1890-1980), in the Suquamish Museum Archives, also in the Seattle Public Library, and in a 1929 book, features Virginia Adams and her white spitz dog “Jumbo”, often attributed to be one of the last woolly dogs (*57*, *58*). However, Leechman wrote in a 1929 report that Mrs. Adams said, “Jumbo is like them [wool dogs], but is a white man’s dog” (*59*).

Two photographs in the Ian McTaggart-Cowan Fond Collection at the University of Victoria attributed to anthropologist Diamond Jenness (1886-1969), show a dog with floppy ears (*60*) in one and another photo in which the ears are obscured (*61*). Jenness and William Henry Arnold “Billy” Newcombe (1884-1960) corresponded with zoologist Glover Allen (1879-1942), the author of the 1920 book *Dogs of the American Aborigines* (*62*) about these photographs. In a letter dated Dec 28, 1935, Jenness quotes Glover Allen’s suggestion that erect ears are “a rather characteristic trait of the Indian dogs so far as I have seen them” (*63*). This statement leaves open the question if one or both Jenness photographs indeed are of wool dogs (*63*).

Introduction to Mutton & the Semiahmoo Bay (SB) Dog:
In the early 2000s, specimens of Mutton and the Semiahmoo Bay (SB) Dog were independently rediscovered by historian Candace Wellman and Russel Barsh. As Barsh described (*13*), both were researching specimens collected for the U.S. National Museum (the precursor to the Smithsonian Institution) in the 1850s by American naturalist C.B.R. Kennerly and American ethnologist George Gibbs. Gibbs and Kennerly were both part of the Northwest Boundary Survey for the United States government.

USNM 4762, “Mutton” – Chiloweyuck Depot

All original tags read: “Indian Dog ‘Mutton’ Chiloweyuck Depot G. Gibbs” and the original packing slip is written: “Mr G[ibb]’s dog ‘Mutton’ Chiloweyuck Indians.”

It is unclear which exact community and location Mutton was originally from. Between 1857-1859, Kennerly spent time collecting natural history specimens in southwest British Columbia. “Chiloweyuck Depot” was a forward camp (*64*, *65*). Today, the town of Chilliwack is on the Fraser River, about 75 km east of Vancouver, British Columbia, and is inhabited by the Stó:lō Nation, a political amalgamation of eleven distinct but closely connected communities whose collective territories extend westward along the Fraser River from the southern point of the Fraser Canyon (Hope) and along the Fraser Valley as far as Langley, and including Chilliwack (*16*, *66*). Gibbs also spent time there with Kennerly, and Mutton may have come from a nearby Coast Salish community, such as the Stó:lō (*16*, *66*).

On August 19, 1859, Kennerly wrote to Spencer Baird, the first curator of the Smithsonian Institution:

> “We got another splendid goat skin which was sent to Camp Skagit where Mr. Gibbs happened to be & he took charge of it; but most unfortunately his famous Indian dog “Mutton” got at it and ate the head off. He sent it to me yesterday & when I opened the bag & saw the injury I could almost have cried. Mutton was sheared a short time ago, & as soon as his hair grows out we will make a specimen of him.” (*67*).

Mutton has a long, very dense double coat with a dense undercoat and long, fine guard hairs. His coat is not pure white but has slightly yellow undertones. His rear and his tail are discolored a copperish red. According to Baird’s directions (circa 1848) for collecting Natural History specimens and objects, mammals “larger than a rat” should be skinned, and the interior of the animal specimens treated with arsenic powder or arsenic mixed with water and alcohol. If arsenic is not available, the skin should be salted down in casks. The skins should be completely dry before being packed away (*68*).

Mutton has small ears in the shape of equilateral triangles, and a very short, pointed muzzle with a small black nose. His lips and paws are black in color. His limbs are relatively large for his size, and his feet are large and wide, especially when compared to the SB Dog. Although woolly dogs had been reported to have perky upright ears and curled, spitz-like tails (*13*), it is impossible to tell if Mutton had these features, given the dry and stiff preservation condition (**fig. S2**). During specimen preparation, his skin was nailed flat to dry – iron nails were left embedded around the jaws and the upper right portion of his neck (**fig. S4**). Mutton was not left to dry completely before being folded and packed away – consequently, his head is permanently folded over onto his back (**fig. S4**). The carpals, phalanges, and paws had been left intact. The paws and toenails look healthy with no visible pathologies, and Mutton does not have double dew claws.

Dimensions of pelt:
70-72 cm from nape of neck to base of tail
Hind leg 19 cm from back edge of back pad to top of leg
Tail length to bone tip 16-17 cm
Hair beyond tip of bone 13 cm

Measurements of hair:
Tail guard hairs 13 cm
Center of back guard hair 10 cm
Center of back under coat ~4 cm
Flank guard hair ~8 cm
Flank undercoat ~3.5 cm

Measurements from X-rays of Mutton's carpals and phalanges (**fig. S4**) suggest that he may have been larger than archaeological woolly dogs (**fig. S7**). It is unknown whether Mutton's size is typical for woolly dogs, if his admixed ancestry affected his size, or if zooarchaeological analyses have not yet captured the breadth of size variability in woolly dogs.

USNM 3512, "Semiahmoo Bay village dog" (SB Dog) – Washington Territory
Tag says '"Indian dog" collected by A. Campbell and C. Kennerly'.

Between 1858 and 1859, Kennerly shipped two dog pelts and a skull to the U.S. National Museum. On March 5, 1858, he wrote to Spencer Baird from Semiahmoo Bay (located today near Blaine and the Lummi Indian Reservation in Washington State, USA):

> "… I had two nice skeletons of the otters, & packed them in a box with weights on the top, & intended to clean them in the morning when to my horror & chagrin the abominable Indian Dogs during the night got out the bones & gnawed them to pieces. In pay for this a beautiful skin of a large woolly Dog now hangs outside in a state of preparation for the Smithsonian Museum & as a warning to all others that may come around here without their owners with them." (*67*).

Barsh explains that Kennerly likely mistook a "village dog" for a woolly dog in his letter to Baird (*13*). This skin was originally assigned field number 106 and is now cataloged USNM# 3512. Barsh describes the SB Dog as a medium-sized dog with a relatively long, uniformly tawny coat, and the undercoat does not match the woolly dog material in the Smithsonian's 19th century Coast Salish weavings in color or texture (*13*). The SB dog is larger in size than Mutton, and superficially resembles an Irish setter, with a long and silky tawny/ochre/reddish coat, a relatively long muzzle, and long, slender limbs (**fig. S3**). The dog's feet are smaller and more delicate than Mutton's. The carpals, phalanges, and paws had been left intact (**fig. S3**). The paws and toenails look healthy with no visible pathologies, and the dog does not have double dew claws.

Dimensions of pelt:
155 cm long
47 cm wide

AL3194 - Port au Choix, Newfoundland
The Port au Choix archaeological site is located on the Port au Choix peninsula, projecting into the confluence of the Gulf of St. Lawrence and the Strait of Belle Isle, on Newfoundland's northwest coast. The area includes several well-preserved sites, including a Maritime Archaic burial ground (Port au Choix-3) with over 100 preserved burials (Port au Choix-3, Locus II), which was excavated from 1967-1969 by Memorial University of Newfoundland (*69*, *70*). The

Maritime Archaic are Indigenous groups in the Atlantic Provinces, dating from approximately 9,000-3,500 years ago, and the burial ground at Port au Choix is thought to date to approximately 4,400-3,300 years ago (*71*). The remains of four Large or "Common Indian" size dogs were recovered from the Port au Choix-3 burial ground (reviewed in (*72*)). AL3194 is an older male, likely weighing between 45-55 pounds, and killed by a blow to the head. The dog was also buried with another dog in a multi-human burial (*73*). The direct radiocarbon dating of the dog is 4,300-3,750 calibrated BP (UCIAMS159456). These dogs at Port au Choix were likely used as companions, hunting aids, or travois dogs (*72*, *73*).

ALAS_015 – Teshekpuk Lake, Alaska (Collection ID: 28769)

This sample (p2 premolar from the lower carnassial) (**fig. S6)** was provided by the University of Alaska Museum of the North, and sent to the Swedish Museum of Natural History, Stockholm for DNA extraction. Approximately 100 mg of bone powder was collected from the cementum layer, following previously described methods for permafrost bone and tooth samples (*74*). The sample was not directly radiocarbon dated, but mtDNA tip-dating suggests an age interval of 0-9,452 years BP (point estimate 3,763 years BP) (*75*).

II. Methods: X-Ray

After taking tissue samples for DNA isolation, we x-rayed both Mutton and the SB dog pelts to get measurements of the bones in the hind feet and forepaws (**figs. S4-S5**). Because of the stiffness of Mutton's pelt and the thickness of his hair, it was impossible to get measurements without using x-ray. The measurement for metatarsal IV of Mutton compared to archaeological "woolly" dogs are in **fig. S7**. We used a PXS5-927EA Microfocus X-Ray Source with a MARS-1717V Digital X-Ray Detector. The X-Ray detector has an imaging area of 3072 x 3072 pixels, with a pixel Size of 139 microns. The spatial resolution is 3.9l pm – 22l pm (Microfocus).

III. Methods: Portable X-ray fluorescence spectroscopy (p-XRF)

To determine what preservatives were present in the pelts of both Mutton and the SB dog, we performed p-XRF analysis. The instrument used was a Bruker Tracer III-SD (handheld p-XRF spectrometer) with a rhodium tube, no filter, no vacuum/helium flush, with an excitation voltage of 30 kV, a current of 30 µA, and a 60s acquisition time. When taking the measurements, the spectrometer was held on a tripod within a couple of millimeters away from the sample surface.

The SB dog and Mutton's pelt XRF analysis highlighted the presence of elements (mainly arsenic, but also chlorine, mercury, antimony, lead, etc.) consistent with previous preservation treatments such as but not limited to mercuric chloride, vermillion, arsenic soap, and orpiment. Amounts vary from one location to another possibly due to multiple applications and the way they were applied. The red stains noticeable on Mutton's pelt contains high levels of mercury (**fig. S8**). Overlay of XRF spectra (**fig. S8**) of the two pelts on the fur side show a lot of similarities apart from the additional presence of antimony on Mutton's pelt. Higher levels of sulfur on Mutton's pelt could be due to the thicker fur and/or additional preservation treatments. Results are summarized in **Tables 1** and **2** below and as well as XRF spectra (**fig. S8**).

IV. Methods: Genomic analyses

Sampling – NMNH

Destructive sampling permissions was obtained from the Division of Mammals, NMNH. To extract DNA from Mutton and the SB dog, samples were collected. Nitrile gloves were worn and sterile techniques were used including bleaching work surfaces and all tools prior to use. Two or three samples were taken from each specimen from different parts of the pelts:

Mutton:
1. Skin clip between hind limbs ~1 cm long in four pieces
2. Cartilage and adherent muscle from inside of right ear pinna, about 10 pieces largest ~5 mm x 8 mm
3. Skin from front right paw near metal tag w/pink string, ~1 cm in two pieces

SB Dog:
1. Skin surrounding lips and nose, ~10 pieces each about 2 mm x 4-5 mm
2. Front right paw had metacarpals exposed, sampled tissue between bones and also some skin clips ~8 pieces of tissue 1mm x 5 mm, skin 3 mm x 8 mm.

Approximately 50-100 mg of skin or tissue was collected for each subsample. Individual subsamples were placed in a 15 mL falcon tube, sealed, and transferred to the NMNH Ancient DNA laboratory.

DNA Extraction, Library Prep, Sequencing – NMNH – Mutton, SB Dog

All ancient DNA lab work on the Mutton and the SB Dog samples was undertaken in the ancient DNA facility at the Smithsonian National Museum of Natural History under accepted protocols for ancient DNA stringency (*76*). DNA was isolated from Mutton and SB Dog tissues using a standard protocol for degraded DNA from soft tissues. Briefly, tissue was agitated overnight at 55°C in a buffer containing CaCl, SDS, DTT, EDTA, and Proteinase K, according to (*77*). The following day, an additional equal volume of proteinase K was added to complete tissue digestion. Following (*78*), 13 volumes of Qiagen buffer PB was added to the lysate, the mixture was passed through Qiagen MinElute columns, washed twice with Qiagen buffer PE, and eluted in 70 µL of TE buffer with tween in two rounds of elution with 15 minutes incubation at 37°C between adding buffer and centrifuging to elute.

Considering the potential damage during previous preservation treatment, the libraries were built by single-stranded library preparation (*79*) with dual indexing (*80*). This construction not only targets double-stranded DNA, but also builds libraries from single-stranded DNA templates, which would potentially retain higher complexity compared to conventional double-strand DNA-based library construction. The concentration of adapters, reagents, and PCR cycles were decided based on double strand DNA input (7x PCR cycles for Mutton and 14x PCR cycles for the SB Dog). Libraries were sent to Admera Health and sequencing was performed using paired 150bp reads on an Illumina HiSeq X10 system. A table with a breakdown of the tissues and extracts that delivered the sequencing data can be found in **DataS1**.

DNA Extraction, Library Prep, Sequencing – CPH/London/Oxford – MU_NP50A_1; AL3194 (Port au Choix dog)

A ~2x coverage sequence of the ancient domestic dog AL3194 was originally published (*3*) but has been re-sequenced at a higher coverage for this publication. DNA was extracted and processed from a pars petrosa in the ancient DNA laboratories at the Globe Institute, University of Copenhagen. Initially the bone was decontaminated for 10 min in a 7% hypochlorite solution. It was next digested in an EDTA, urea and proteinase K buffer as in (*81*), the digest was purified using phenol-chloroform (*82*). The original libraries that were previously published (*3*) were re-indexed as previously described (*3*). In short, Illumina libraries were built according to (*83*) and a six base-pair barcode joined to the adapter, creating an "internal adapter" resulting in double-barcoded libraries. The single-end, 80-bp libraries were then sequenced on an Illumina HiSeq 2500 at the Danish National High-Throughput Sequencing Centre (Copenhagen) and on an Illumina NextSeq 500 at the Natural History Museum (London), respectively.

DNA Extraction, Library Prep, Sequencing – SMNH – ALAS_015

ALAS_015 is a domestic dog excavated from Teshekpuk Lake, Alaska. The sample was processed and sequenced alongside multiple samples for a previous publication (*75*) at the Swedish Museum of Natural History in Stockholm, Sweden, using previously described methods for permafrost bone and tooth samples (*74*). In brief, this involved DNA extraction using the methodology previously described (*74*) and double-stranded Illumina library preparation as described (*83*) with dual unique indexes and the inclusion of USER enzyme. Between eight and ten separate PCR reactions with unique indexes were carried out for each sample to maximize library complexity. The libraries were across three Illumina NovaSeq 6000 lanes with an S4 100-bp paired-end set-up at SciLifeLab in Stockholm.

Sampling, DNA Extraction, Library Prep, Sequencing – UC Davis – coys19 (modern coyote)

Coys19 (S19-1195) is a modern coyote (*Canis latrans*) from Goshen County, Wyoming. Frozen muscle tissue was sampled and DNA was extracted using the DNeasy 96 kit according to manufacturer's instructions. The library was constructed and sequenced using a partial lane of an Illumina paired-end 150 base-pair Novaseq 6000 S4 through the DNA Technology Core, University of California, Davis Genome Center.

DNA Extraction, Library Prep, Sequencing – NIH

WGS data was generated from samples collected with owners signed consent in accordance with standard protocols approved by the NHGRI IACUC committee, protocol #GFS-05-1. Saliva samples were owner collected and purified using the Performagene® (PG-100) saliva collection kit (DNA Genotek). Blood samples were collected by licensed veterinarians or veterinary technicians and genomic DNA was extracted by phenol-chloroform extraction. Purified DNA was resuspended in 10 mM Tris, 0.01 mM EDTA, pH 8.0 and stored at -80°C (*84*). Libraries were constructed using Illumina® DNA PCR-Free Prep Kit with 150 bp paired-end inserts. Libraries were sequenced at the NIH Intramural Sequencing Center (NISC) using the Illumina NovaSeq 6000 platform to a target coverage of 20X.

Genome sequence data processing – NMNH

All sequence read data resulting from paired-end sequencing had adapter fragments removed, reads trimmed downstream of the first base with quality score <20, and forward and reverse

reads merged with AdapterRemoval2 (*85*). We aligned the resulting merged and adaptor-trimmed sequences to the dog canFam3.1 genome using BWA *aln* with seed disabled (*86*). Duplicates were removed using samtools rmdup and reads were then filtered using samtools with a length of at least 30 base pairs and a mapping quality of at least 20. Reads were re-aligned around short indels using GATK version 3.8.0 (*87*). Post-mortem damage was quantified using mapDamage 2.0 (*88*), yielding very low deamination as expected with 19th century specimens **(figs. S9-S10)**.

Genome sequence data processing – CPH

All data generated data from the Port au Choix dog constituted single-end sequencing. The raw fastq files for the previously published sequences from the sample (NCBI: ERR2061050) and the newly generated sequences from the sample were trimmed of adapters with AdapterRemoval2 (*85*). Subsequently the data was aligned to the canFam3.1 genome with using BWA *aln* but with seed disabled. PCR duplicates were then removed using MarkDuplicates by picard (http://broadinstitute.github.io/picard).

Genome sequence data processing – SMNH

The genome processing was performed according to methods previously described (*89*). The adapters were trimmed and paired-end reads merged using SeqPrep v1.131 with default settings and a minor modification in the source code, allowing for the best quality scores of bases in the merged region (*90*). Sequencing reads were merged and mapped against the reference mtDNA genome for the domestic dog (canFam3.1) using BWA (*86*) *aln* with default settings and deactivated seeding (-l 16,500), allowing more substitutions (-n 0.01) and allowing up to two gaps (-o 2). BWA samse was used to generate alignments in SAM format. Resulting reads were processed in Samtools v1.933, converted to BAM format, sorted, and indexed. Duplicates were removed from the alignments using a custom python script to avoid inflation of length distribution for loci with deep coverage (*86*). Picard v1.141 (http://broadinstitute.github.io/picard) was used to assign read group information including library, lane, and sample identity to each bam file. Reads were then re-aligned around indels using GATK v3.4.0 34 (*87*) and reads with mapping quality 30 were kept.

Genome sequence data processing – NIH

Raw FASTQs were aligned to CanFam 3.1 using BWA mem (*91*) and sorted with Samtools. Base quality score recalibration and duplicate marking were applied to each sample (*87*, *92*), and Haplotypecaller was used for variant discovery (*93*). Variant calling was performed using GATK4 best practices (*92*).

Error estimation in ancient genomes

For the 40 ancient nuclear genomes analyzed here (**DataS1**), we used ANGSD (*94*) to estimate sequence error rates in aligned reads following the method described in (*95*). As expected, deamination drove higher observed mismatch rates in C->T and G->A mismatch types, which are mitigated as described below. We observed very low error rates across other mismatch types: mean error = $3.95 \cdot 10^{-4}$, range $1.32 \cdot 10^{-4}$ to $1.15 \cdot 10^{-3}$. The highest error rates by mismatch type were observed in C->A and the complementary G->T (mean $6.4 \cdot 10^{-4}$, range $1.4 \cdot 10^{-4}$ to $2.0 \cdot 10^{-3}$ in both types). Overall, we observe low error rates in ancient genomes, and no outliers with

problematic levels of sequencing error. Overall and per-mismatch error rates are given for all samples in **DataS1**.

Damage mitigation and variant calling in ancient specimens

Because we used ancient dog datasets from a wide variety of studies with variable DNA preparation and data handling strategies, we adopted a conservative approach to variant calling in light of cytosine deamination in ancient DNA. We first used mapDamage (*88*) to independently model C->T and G->A misincorporation in both forward and reverse positions, accounting for all permutations of single- and double-stranded library preparation and adapter configurations. We then used the delta-S and lambda values inferred in all four contexts to rescale base quality scores using the phred scale to enforce observed uncertainty in possibly deaminated bases according to their position, and discarded reads with length <30bp. For heterozygosity and ROH estimation in Mutton and the Port au Choix dog (see below), we used a version of the bam alignment files without base rescaling, as ROHan includes its own integrated strategy for error mitigation and these analyses were based on transversions only. We then used samtools *mpileup* (http://www.htslib.org/doc/samtools-mpileup.html) to summarize all positional read support with base quality recalibration disabled, and with a minimum base quality of 20 after rescaling for damage. We finally created a genome-wide pseudohaploid fasta file—we selected a base at random for each position from an allele supported by ≥2 non-redundant reads, and with maximum coverage at the individual's .999 quantile, which was sufficient to avoid spiking coverage artifacts. Pseudohaploid base calls were extracted from these ancient dogs to match the modern reference panel using bedtools (*96*) and merged with modern reference panel using PLINK (*97*). SNP-based analyses (d-, outgroup-*f3* and *f*4-ratio statistics) were restricted to 14.45 million sites with minor allele frequency ≥0.01 and genotype missingness ≤0.5.

Bayesian molecular clock mitochondrial genome phylogeny

Bayesian phylogenetic analyses were computed using BEAST v2.6.3 (*98*). We used tip dating, the strict molecular clock and a lognormal distribution with a mean in real space of $1.0\cdot10^{-8}$, an upper bound of $1.0\cdot10^{-6}$ substitutions/site/year, and a lower bound of $1.0\cdot10^{-10}$ substitutions/site/year (these bounds are part of a separate uniform prior and are not part of the lognormal distribution itself). HKY+Γ substitution model was used with four rate categories for gamma-distributed rates across sites. An exponential prior for kappa and a lognormal prior was selected for the gamma shape prior, with default parameters. These priors were previously used in the BEAST v2 analyses on ancient and modern dog mitochondrial genomes (*99*). Mean date estimates for all the mtDNA sequences for the analysis were used because accounting for age uncertainty has negligible or minimal impacts on the resulting estimates in AL3194 (*99*). Constant coalescent population model was selected as the tree prior. Default settings were used for all other parameters. Posterior distributions of parameters were estimated by Markov chain Monte Carlo (MCMC) sampling. Samples were drawn every 10,000 steps over a total of at least 1 billion steps. The first 15% of samples were discarded as burn-in. Sampling was considered sufficient when the effective sample size of each parameter exceeded 100. When required, additional MCMC analyses were run to achieve sufficient sampling. The trace files were assessed using Tracer (*100*) and samples from two independent runs were merged using LogCombiner (*101*).

*Ancestry analyses: outgroup-*f3 *statistics*

To reinforce the PCD ancestry of Mutton and to explore whether Mutton has any European ancestry, we calculated outgroup-*f3* statistics using AdmixTools v7.0.2 (*102*). Outgroup-*f3* statistics were calculated for Mutton, SB Dog, Port au Choix dog (AL3194) and Weyanoke dog (AL3223), comparing each respective dog to 229 other ancient and modern dogs, and GrayFox as the outgroup population (**fig. S17**). *f*3(GrayFox; Mutton, B) reveals that Mutton has the highest *f*3 value and genetic similarity with other PCD dogs, specifically the 4,020 year old dog from Port au Choix, Newfoundland and the 1,000 year old dog from Weyanoke Old Town, Virginia, relative to the outgroup Gray Fox. The *f*3-(AL3194 PortauChoix, B, GrayFox) and *f*3-(AL3223 Weyanoke, B, GrayFox) analyses also reinforce the greatest similarity to the PCD dogs, followed by two ancient Arctic dogs from Alaska (ALAS_015) and Zhokov Island in the East Siberian Sea (CGG6) (**fig. S17**). As for the SB Dog, the outgroup-*f3* statistics have greater error bars because of lower coverage, but the dog shows greatest similarity to ancient dogs from Northwest and Arctic Siberia (TRF.05.17 and TRF.05.16) and Alaska (ALAS_015) (**fig. S17**).

Ancestry analyses: D-statistics

We calculated D-statistics using AdmixTools v7.0.2 (*102*). D-statistics provide evidence for admixture and gene flow. The syntax is: (W, X, Y, Z), where W is GrayFox, X is a modern dog breed, Y is Port au Choix (AL3194) or Weyanoke dog (AL3223), and Z is Mutton. If the Z-score is positive, then the gene flow occurred between X and Z, assuming W is a true outgroup. If the Z-score is negative, then the gene flow occurred between X and Y. The results suggest evidence of recent European admixture in Mutton (Z-score > 3), with highest Z-scores coming from admixture sources of boxers, Portuguese water dogs, English Cocker Spaniels, and Lagotto Romagnolo breeds. Moreover, there is a positive correlation between D-statistic values and the Z-scores, of both Port au Choix dog and Weyanoke dog, relative to Mutton.

Ancestry analyses: f4-ratio tests

To model the Mutton's ancestry, we used *f4*-ratio analysis with the following syntax: *f4*(A, Out; Mutton, AL3194 PortauChoix): *f4*(A, Out; B, AL3194 PortauChoix) where modern dog breeds are in the A and B placement, and AL3194 (Port au Choix dog) serves as a proxy for all ancient PCD dogs. We used modern dogs for the donor placements because ancient European dogs have too much admixture signal from ancient Arctic dogs, where it cannot be distinguished whether PCD dogs have Arctic ancestry or recent Arctic admixture (*19*, *103*). Moreover, the modern dogs are a better proxy for what European settlers would have brought than ancient, multi-kya dogs. Six modern dog breeds selected are: Chinese Crested dog, English Cocker Spaniel, Dalmatian, German Shepherd, Lagotto Romagnolo, and Portuguese Water Dog (**fig. S18**). These dogs were chosen because when performing D-statistics, these modern dog breeds had the highest Z-score > 3 when in the admixture source placement X (GrayFox, X, AL3194 PortauChoix, Mutton), indicating gene flow between X and Mutton (**DataS1**).

Ancestry analyses: DATES

To estimate the timing of European admixture into Mutton's predominately PCD ancestry, we used DATES (*104*) to analyze the distribution of chromosomal ancestry blocks. We used assumed 1Mbp = 1cM and used default settings with jackknife estimation of standard error by reiteratively leaving out one chromosome. The PCD population was represented by the Port au Choix dog—the only high-coverage PCD genome currently available—and the European source population was represented by 27 individuals across the same six breeds used above in *f4*-ratio

tests. In our case, the precision of this method is limited due to the scarcity of high-quality PCD source population data, and the likely recency of admixture. However, our estimate of admixture 10.8 generations in the past ± one standard error of 4.9 generations is broadly consistent with post-colonial admixture from one or more European dogs in Mutton's background.

dN/dS selection analyses

An elevated ratio of non-synonymous (dN) to synonymous (dS) substitutions in coding regions can indicate selection on a basis of a single individual, and so offers insight into woolly dog selection pressures based on Mutton. Working within a single target genome with variable coverage among genes, we are very limited in our ability to identify selection via statistical dN/dS outliers. That is, we cannot rule out elevated dN/dS specific to Mutton's lineage in genes by chance through functionally neutral mutation and drift. Nonetheless, this strategy provides a starting point for interrogating plausible interaction between genetic loci and woolly dogs' unique phenotype, and yielded compelling links to several wool-, skin-, and hair-related loci from previous literature (see below).

The branching order used in dN/dS analysis to identify genes under selection in Mutton compared to other canids was used according to (*50*). We separately estimated dN/dS in Mutton and three other dogs—a boxer to represent European dogs, a New Guinea singing dog representing the Sahul lineage (*19*), and the Port au Choix dog (AL3194) representing PCDs. In each gene alignment, we hard-masked all sites there were missing in at least one genome so that results would not be biased by variable genomic coverage. We analyzed 11,112 genes for high dN/dS ratios, restricting analyses to genes with at least 100 codons called in all individuals. Following previous studies (*105*, *106*), we accommodated high gene-level stochasticity in dS by first calculating a single genome-wide dS_{genome} value for each lineage, and then estimating dN_{gene}/dS_{genome} at all loci independently. Following previous studies (*105*, *106*), we restricted analysis to loci where local dS ≤ the mean plus 2 standard deviations of genome-wide dS, and considered genes with $dN/dS_{genome} > 1.5$ to be the strongest positive selection candidates. However, we further restricted our identification of selection candidates in Mutton to genes where $dN/dS_{genome} = 0$ in the other three dog lineages. This helps us assume that inferred selection most likely reflects woolly dogs' background, compared with selection on PCDs or even selection associated with dog domestication. This approach yielded 125 candidates for selection, as detailed further in the main text.

Following the methodology described in (*107*), we also calculated gene-level ratios of nonsynonymous to synonymous polymorphisms (pN/pS) in a sample of 95 modern dog genomes (**DataS1**). The goal of this analysis is to test whether our dN/dS_{genome} approach with a standardized denominator may enrich for genes that tend to tolerate polymorphism, leading to a biased set of selection candidates or likely false positives. After (*107*), we first examined the effects of all possible single mutations in the alignment on amino acid identity to quantify the number of potential synonymous and non-synonymous mutations under a uniform mutation model. Polymorphic sites between all pairs of samples were then assessed as synonymous or non-synonymous, so single values of observed/potential polymorphisms could be computed for both synonymous (pS) and non-synonymous (pN) mutations. The ratio of these values, pN/pS, can be treated as a proxy for tolerance of amino acid substitutions at the gene level. Comparison of pN/pS values between the 125 selection candidates and all other genes revealed no significant

difference (Wilcoxon p = 0.134; Students t-test p = 0.174). On this basis, we observe no biasing effect on tolerance of polymorphism in selection candidates introduced by the dN/dS_{genome} approach.

Gene Ontology

We used the GO database within g:Profiler (*108*) to identify any functional category enrichment among the set of 125 genes within the woolly dog lineage dN/dS_{genome} values >1.5, *Canis lupus familiaris* as the query organism, and all known genes for the statistical domain scope. We found significant enrichment following the g:SCS algorithm (*108*) of multiple test corrections, which is calculated based on a P-value of 0.05. This algorithm operates under the assumption that genes associated to a given GO term are implicitly associated to all the general parents of this term, since GO consists of hierarchically related general and specific terms. Genes were significantly enriched in 5 GO: Molecular Function categories (calcium-dependent phospholipid binding, molecular function, transferase activity, catalytic activity, ion binding); 3 GO: Biological Process categories (regulation of cellular processes, multicellular organismal process, biological_process); 3 GO: Cellular Component categories (cellular_component, cellular anatomical entity, membrane); and 2 KEGG categories (KEGG root term, Metabolic pathways); and 1 Human Phenotype Ontology category (Autosomal recessive inheritance) (**fig. S11**). Many individual genes were found within multiple GO functional categories (**DataS2)**.

We used the hypergeometric test in analyzing gene enrichment in GO categories using GOfuncR (*109*). The hypergeometric test compares positively selected genes in Mutton's lineage compared to "background" genes that are conserved in all canids. GO annotations and gene coordinates were used using the *Homo sapiens* annotation package. Correction for multiple testing and test interdependency was computer using family-wise error rates (FWER), which are based on random permutations (1000 random datasets) of the gene-associated variables. The results for from both tests are in **DataS2**, "res_Hypergeometric" tab. Categories involving cell signaling and cell metabolism are generally enriched (overrepresented raw $p<0.01$). No GO category containing the terms "hair cycle" or "skin" are overrepresented (raw $p>0.01$).

Annotation of candidate genes under selection

To home in more specifically on the gene candidates that may contribute to the woolly dog phenotype, we used DAVID for initial functional annotation, and additionally manually annotated the candidate genes through a literature search. The provided gene list comprising 125 genes is in **DataS3** (*110*, *111*), "Annotations" and "geneList" tabs.

Within these 125 genes, through manual curation we identified 28 genes as candidates involved in the hair growth cycle of woolly dogs (**fig. S12**). We determined that manual curation was necessary due to the limitations of GO category databases in adequately identifying up-to-date gene associations published in the literature. Our assessment is reflected through querying several genes on Gene Ontology Resource (http://geneontology.org/) which in the main manuscript we have identified as related to woolly hair and skin – *KANK2*, *PCOLCE2*, *KRT77*, *GPNMB*, *CERS3*, and *ANXA4*. The results are listed in **DataS2**, "AmiGO2" tab.

Key words related to hair and skin do not appear in any of the GO descriptions for *KANK2*, *PCOLCE2*, *GPNMB*, and *ANXA4*. Key words for skin do appear in the GO descriptions for *KRT77* ("structural constituent of skin epidermis" and "keratinization") and *CERS3* ("cornification" and "keratinocyte differentiation") however all GO terms are dominated by more non-specific molecular, cellular, and structural processes (e.g. "protein binding", "cytoplasm", "cell adhesion", "DNA binding", "calcium ion binding").

In addition, we have queried Gene Ontology Resource and MGI database (http://www.informatics.jax.org) 15 hair-related genes (keywords "hair cycle" or "woolly hair") in found in the literature. The genes queried are: *AHNAK2* (*25*), *KRT8* (*25*), *FLG* (*25*), *PRSS8* (*25*), *P2RY5/LPAR5* (*112*, *113*), *LSS* (*114*), *C3ORF52/BC016579* (*115*), *LIPH* (*116*), *LHX2* (*117*), *FGF1* (*118*), *FGF2* (*118*), *FGF5* (*118*), *DKK2* (*118*), *NOCTUM* (*118*), and *AXIN2* (*119*). With the exception of *FLG* with associated GO categories of "cornified envelope", "epidermis development", "epidermal cell differentiation", "establishment of skin barrier", among other terms, and *KRT8*'s association with "keratin filament*",* most of the GO terms are dominated by non-specific molecular, cellular, and structural processes (e.g. "intermediate filament", "cytosol", "protein binding", "centrosome") or more specific processes not clearly related to skin or hair (e.g. "ubiquitin protein ligase binding", "negative regulation of canonical Wnt signaling pathway", "beta-catenin binding", and "positive regulation of sodium ion transmembrane transport"). These results are listed in **DataS2**, "LitHairGenes_AmiGO2".

Finally, additional query results in **DataS3**, "MGI_GO_MP_Databases" tab demonstrate that panels of hand-picked hair genes from the literature do not flag these categories as enriched. We queried the 125 genes under positive selection against several GO and mammalian phenotype lists in the MGI database. These 125 genes under positive selection had each been queried against the genes within 3 GO terms (SkinDevelopment GO:004358, 340 genes; HairCycleProcess GO:0022405, 121 genes; HairCycle GO:0042633, 142 genes) and 2 MP (Mammalian Phenotype) terms (IntegumentPhenotype MP:0010771, 6,991 genotypes; abnormal coat/ hair morphology MP:0000367, 3,260 genotypes). SkinDevelopment GO:004358 contained 3 genes out of 125, IntegumentPhenotype MP:0010771 contained 10 genes out of 125, and abnormal coat/ hair morphology MP:0000367 contained 2 genes out of 125. HairCycleProcess GO:0022405 and HairCycle GO:0042633 contained 0 genes out of the 125 queried. By going by this assessment, only 14 individual genes are associated with skin development, mammalian integument (which includes hair), and abnormal coat/hair morphology.

The hair follicle is a dynamic environment that is continuously remodeled (*120*). Hair is formed by rapid cell division and differentiation of stem cells that form keratinocytes that migrate, flatten, and die, forming dead, keratinized cells (*121*). The final hair product exposed on the surface of the skin is composed entirely of keratin (dead cells). Hair follicle growth is regulated in a cyclical manner, with stages of rapid growth and elongation of the hair shaft and periods of quiescence and regression. In the hair growth cycle, hair follicles undergo anagen, where an entire hair shaft is grown from tip to root; catagen, where hair stops growing and the hair follicle undergoes apoptosis-driven regression; telogen, a rest phase where the follicle prepares its stem cells to receive a signal for the next growth phase; and exogen, where the entire shaft is released. The events of the hair growth cycle is complex and involves tight regulation of stem cell quiescence and activation, cell proliferation, differentiation, and apoptosis (*122*).

Several genes with potential links to the unique woolly hair phenotype include *KANK2*, *KRT77*, and *GPNMB* which are discussed in the main text. As discussed in the main text, Mutton contains a mutation in the *KANK2* gene immediately adjacent to a causal variant in humans linked to a congenital “woolly” hair phenotype (*32*). This substitution observed in Mutton is unique among the canids. In pairwise comparisons, the *KANK2* amino acid sequence is 89.5% conserved between dogs and humans, 99.4% conserved on average between all canids used in the dN/dS analysis, and 99.85% conserved on average the dogs used for pN/pS analysis.

We also identified twenty genes associated with cell replication and proliferation, or cytoskeletal components: *ARL14EP* (ADP ribosylation factor like GTPase 14 effector protein) (*123*), *CDIPT* (CDP-diacylglycerol--inositol 3-phosphatidyltransferase) (RefSeq, Nov 2013), *CENPQ* (centromere protein Q) (*124*), *CFAP36* (cilia and flagella associated protein 36) (*125*), *CGRRF1* (cell growth regulator with ring finger domain 1) (*126*), *DNAAF3* (dynein axonemal assembly factor 3) (RefSeq, May 2012), *FOSL1* (FOS like 1, AP-1 transcription factor subunit) (RefSeq, July 2012), *KATNAL1* (katanin catalytic subunit A1 like 1) (Alliance of Genome Resources, Apr 2022), *KCTD21* (potassium channel tetramerization domain containing 21) (*127*), *KLHL22* (potassium channel tetramerization domain containing 21) (Alliance of Genome Resources, Apr 2022), *LOC100682940* (putative speedy protein E7) (*128*), *PNMA2* (PNMA family member 2) (Alliance of Genome Resources, Apr 2022), *QSOX1* (quiescin sulfhydryl oxidase 1) (RefSeq, Jul 2008), *RANBP10* (RAN binding protein 10) (RefSeq, Feb 2016), *SART1* (spliceosome associated factor 1 recruiter of U4/U6.U5 tri-snRNP) (RefSeq, July 2008), *TJP3* (tight junction protein 3) (RefSeq, May2022), *TOB1* (TOB1 transducer of ERBB2, 1) (RefSeq, Aug 2011), *TRIT1* (tRNA isopentenyltransferase 1) (RefSeq, Aug 2015), *TTC23L* (tetratricopeptide repeat domain 23 like) (Alliance of Genome Resources, Apr 2022), *WEE1* (WEE1 G2 checkpoint kinase) (*129*).

The extracellular matrix (ECM) is a large network of proteins and other molecules that encompasses and gives structure to cells and tissues, allowing for cell communication, growth, movement, proliferation, adhesion, differentiation, and apoptosis. The ECM also provides an important role in tissue damage repair (*130*). During anagen development, the ECM increases rapidly and decreases when the transition to full anagen is complete (*120*). We identified three genes linked to the formation of extracellular matrix (ECM) components: *PRDM5* (PR/SET domain 5) and *HAPLN1* (hyaluronan and proteoglycan link protein 1) which both encode for genes involved in ECM development and maintenance (*28*), and *PCOLCE2* (procollagen C-endopeptidase enhancer 2) which enables collagen and heparin binding activity, and is downregulated in growing hair follicles (*131*).

The development of the hair follicle requires the presence of blood vessels that nourish the growing follicle, supporting the delivery of nutrients and the removal of waste (*121*). We identified three genes associated with the vascular system and the mediation of blood pressure, including *GPR180* (G-protein coupled receptor 180) (*132*), *PLVAP* (plasmalemma vesicle associated protein) (*133*), and *AGT* (angiotensinogen) (*134*).

Finally, we identified three genes associated with the skin or epidermis: *GPNMB* (described in the main text), *ANXA4* (annexin 4) (*135*, *136*)), and *CERS3* (ceramide synthase 3), which is

responsible for creating a protective barrier from the environment in the epidermis. *CERS3* mutations cause autosomal recessive congenital ichthyosis in humans (*27*).

Variants associated with coat color

We checked Mutton's genotype of 15 different variants associated with coat color and texture variation in dogs, summarized in **Data S4**. Although these sites are covered by a small number of reads (up to 7) manual examination of curated read alignments at these sites provides evidence of at least one allele in most cases.

FGF5 genotypes

There are 5 known polymorphisms in the *FGF5* gene (fibroblast growth factor 5) that are linked to the long hair phenotype in dogs (*33*). The c.284G>T mutation (p.Cys95Phe) is found in most long-haired dogs, although it does not account for long hair in all dogs. Long hair in certain dogs appears to be expressed in a heterogeneous fashion, with multiple alleles present, even within the single *FGF5* gene. For example, the g.8193T>A and c.559_560dupGG mutations have been identified in Afghan hounds (*33*). We investigated whether Mutton's long hair phenotype can be attributed to any of the *FGF5* genotypes conferring long hair. We found that Mutton has only the c.284G>T genotype, with 3 reads covering the position chr32:4509366. Disregarding sequence error and reference bias, 3 consistent reads confer 87.5% chance of a homozygote. All long-hair-associated mutations in dogs follow a recessive mode of inheritance (*33*), so it is reasonable to conclude that Mutton is homozygous for the c.284G>T mutation. However, for other mutations in *FGF5*, Mutton is the wild type (**DataS4**). If there are any other polymorphic alleles responsible for Mutton's long hair, they lie elsewhere.

MC1R genotypes

There are multiple polymorphisms in the *MC1R* (melanocortin 1 receptor) gene, a G-protein-coupled receptor primarily located on the surface of melanocytes. When *MC1R* signaling is induced, melanocytes produce brown-black eumelanin. Mutations in *MC1R* are linked to coat color variation in domestic and wild animals including brindling, spotting, and a melanistic mask or grizzle in dogs (reviewed in (*137*)). The c.916C>T mutation (p.Arg306ter) is associated with a light coat color in Australian Cattle dogs and Siberian huskies. Mutton has the wild type genotype (c.916C) with 3 reads covering the position of chr5:63694334, therefore most likely a homozygote, disregarding sequence error and reference bias. Mutton is also wild type for the 63695679C>G mutation which is associated with a light coat color in Australian cattle dogs (*138*), with 2 reads covering the position of chr5:63695679. A c.816_817delCT mutation in chr5:63694432 confers a light coat color in Alaskan and Siberian huskies (*138*), and there was no coverage in that position in Mutton. Dark spots (e.g. "grizzle" or "domino " patterns in Salukis and Afghan hounds) are linked to a c.233G>T (p.Gly78Val) mutation (*34*, *138*), and Mutton appears to be wild type at the position of chr5: 63695017 with 1 read. c.790G>A (p.Val264Met) is linked to a "black mask" coloring in Leonbergers and Malinois (*138*, *139*), and Mutton appears to be homozygous wild type for that allele, with 3 reads spanning the position chr5: 63694460.

KRT71 genotype

Curly or wire hair coats in the Airedale Terrier, Bichon Frise, Kuvasz, Portuguese Water dog, Poodle, Welsh Terrier, and Wire Fox Terrier breeds are associated with a c.451 C>T

(p.Arg151Trp) mutation in the *KRT71* (keratin 71) gene (*34*). Mutton carries the wild type allele in 2 recovered reads spanning the position at chr27: 2539354.

<u>MFSD12 genotypes</u>

A light coat color is also associated with a c.166 C>T mutation in the *MFSD12* (major facilitator superfamily domain containing 12) gene in Shepherds, Poodles, Cotons de Tulear, Bichon Frise dog breeds (*140*). Unfortunately, we could not genotype Mutton for this variant, as there was no coverage at that position.

<u>MLPH genotypes</u>

Light or diluted coat color is associated with three recessively inherited variants in the *MLPH* (melanophilin) gene. A c.−22G > A mutation is found in beagles and Doberman pinschers (*141*), and Mutton is wild type at this allele, with 3 reads spanning chr25:48121642. The c.705G > C mutation is found in chow chows (*142*) and Mutton is wild type at this allele with 7 reads spanning chr25:48150787. Finally, a c.669C >T mutation is found in many dog breeds (*143*) and Mutton is wild type at this allele with 3 reads spanning chr25:48150751.

Heterozygosity analysis

We used ROHan (*144*) to estimate autosomal genomic heterozygosity in Mutton, the Port au Choix dog, and 89 comparative breeds and village dogs, providing a mappability mask generated with SNPable (https://lh3lh3.users.sourceforge.net/snpable.shtml), and using a --rohmu value of $4\cdot10^{-5}$. In estimating genome-wide heterozygosity, ROHan used the Watterson's theta formula where segregating sites is four times the mutation rate multiplied by the effective population size (*144*). By assuming that the effective population size (Ne) of contemporary grey wolf populations is ~1,000 (*145*), an inbred population will have one order of magnitude lower level of segregating sites and mutation rate of $1\cdot10^{-8}$. Rather than using the damage-rescaled read alignments described above, we used bam2prof in the ROHan package to accommodate the low level of deamination in Mutton's genome, and we restricted the analysis to transversions only in all samples to accommodate the higher deamination in the Port au Choix dog at low coverage. Because of Mutton's relatively low coverage (estimated at 3.44x in ROHan), we tested for possible depression of heterozygosity estimates by randomly downsampling all other dogs to the same level ten times independently and repeating the analysis. Downsampled runs were highly consistent between replicates for each dog (average standard deviation of replicates $1.4\cdot10^{-6}$) and drove a mean 11.2% decrease in heterozygosity estimates. We show the downsampled estimates in **Fig. 3C**, and full results are provided in **DataS1** – ROHanDataset.

Although ROHan is validated for 5-8x coverage for accurately inferring ROH in samples with variable deamination (*144*), it can be used to estimate global heterozygosity in samples with lower coverage, especially with low deamination and/or when analyzing transversions only. ROHan was tested on three levels of post-mortem deamination: 1) high in the "ATP2" sample, 2) medium in "LaBrana", and 3) low in "Ust-'Ishim" (*144*) . These samples have their highest deamination rate at least 0.3, 0.15, and 0.06 respectively. Mutton's low damage rate assessed by ROHan (0.05) is akin to a low damage sample tested in ROHan, Ust-'Ishim, with deamination rate 0.06 (*144*). The estimates of genome-wide heterozygosity using Watterson's theta under a lower coverage (3x) are only slightly lower than the true simulated Watterson's theta estimate (*144*). Because we use transversions only and compare between samples downsampled to a

standardized coverage level, this approach provides a robust estimate of the relative global heterozygosity among samples.

Runs of Homozygosity (ROH)
Because ROHan has not been validated for accurate ROH inference at Mutton's coverage level (*144*) we adapted a low-coverage method for conservative inference of long ROH in ancient genomes (*24*). Briefly, we used the *.hEst.gz output from the downsampled ROHan runs described above as an estimate of heterozygosity in 500kbp non-overlapping windows to standardize across variable coverage levels, using transversions only. Windows with heterozygosity below $4 \cdot 10^{-5}$ and at least 50,000 valid sites were considered candidates for runs of homozygosity. We inferred long runs of homozygosity (at least 2.5Mbp) where at least five consecutive windows met these criteria. The total genomic fraction in long ROH reported in **Fig. 3A** and **DataS1** was computed as the number of total windows in long ROH on this basis divided by the total number of windows with the minimum number of valid sites analyzed. This approach, which has been previously validated for ancient goats (*24*) focuses on providing a conservative and standardized estimate of long ROH for comparison between individuals.

VII. Methods: Stable isotope analysis

Destructive sampling permissions was obtained from the Division of Mammals, NMNH. For $\delta^{13}C$ and $\delta^{15}N$ from bone collagen, samples of ~150 mg were taken from cortical bone, excluding bones with pathological changes on the principle that changes in the metabolic pathways of the tissue as a result of disease may affect the isotopic values (*146*). As both dogs had been processed into pelts by the explorers, few bones remained for sampling. Sample options for cortical bone were limited, however bones in the paws had been left attached to the pelts and were therefore utilized for stable isotope analysis. For both Mutton and the SB dog, a second metacarpal were sampled with a rotary saw. The bone samples were abraded with a Dremel attachment to remove soft tissue, and then prepared following a modified Longin method (*147*, *148*). Samples were demineralized in 0.6 M HCl at 4°C for 24 h increments until reaction ceased, rinsed five times in ultra-pure 18.2MΩ H_2O, then reacted in 0.03 M HCl at 95°C for 18 h to separate soluble and insoluble phases of collagen. The resulting supernatant was lyophilized to isolate purified collagen extract.

For $\delta^{13}C$ and $\delta^{15}N$ from hair keratin, hair was cut as close to the skin as possible. Both dogs appeared to be double-coated, and since these two types of hairs grow at different rates, the undercoat hairs were discarded. The topcoat hairs were cut into incremental 1 cm sections to create a time sequence. In the 19th century, collectors would treat animals with arsenic before shipping (*149*), and the Smithsonian Institution would use arsenic trioxide (As_2O_3) or arsenous acid and mercury as mercuric chloride ($HgCl_2$) as pesticide controls (*150*). To remove these applied treatments, the hairs were soaked in a chloroform/methanol solution for 4 hr. Hair was then rinsed five times in ultra-pure 18.2MΩ H_2O and oven-dried at 40°C overnight.
All samples were analyzed on a Thermo Delta V Advantage mass spectrometer at the Smithsonian Museum Conservation Institute Stable Isotope Mass Spectrometry Laboratory. Collagen and keratin were weighed into tin capsules, combusted in an Elementar Isotope Cube, and the resulting N_2 and CO_2 gases measured for $\delta^{15}N$ and $\delta^{13}C$ values. Data is presented in the standard delta notation where $\delta X = [(R_{sample}/R_{standard}) - 1] * 1000$; where X is the heavy isotope of interest (^{15}N or ^{13}C), R is the isotope ratio ($^{15}N/^{14}N$ or $^{13}C/^{12}C$), the standard is atmospheric air

(N) or V-PDB (C), and units are permil (‰). All runs include a set of reference materials for every 10-12 samples. Reference materials include Costech Acetanilide (calibrated to USGS40 [L-glutamic acid] and USGS41 [L-glutamic acid]) and USGS66 (glycine). Reproducibility of reference materials and in-house keratin and collagen standards is ≤0.2‰ (1σ) for both $\delta^{13}C$ and $\delta^{15}N$; error associated with all sample data points are reported as ±0.2‰.
Although diagenetic alteration is expected to be minimal as these dogs were kept in controlled museum environments, previously determined criteria for well-preserved collagen were nonetheless required for statistical analysis. For bone, a C:N ratio of 2.8–3.6, %C values between 15–47% by weight and %N values between 5–17% by weight (*151*, *152*). For hair keratin, C:N ratios should be between 3.0–3.8 (*153*).

Results

All samples fell within the quality markers of good preservation as listed above; all samples were included in subsequent analyses. To accommodate the known offset between hair keratin and bone collagen stable isotope values, values of 1.41 ‰ and 0.86 ‰ were added to the hair keratin $\delta^{13}C$ and $\delta^{15}N$ values, respectively (*153*). While these values have been derived from humans which may have slightly different metabolic routing compared to dogs, these conversions have been used in previous zooarchaeological analyses of dog isotope values (*154*) and show good agreement in fur and bone from the same dogs when this correction was applied. **DataS5** contains the bone collagen and converted hair keratin $\delta^{13}C$ and $\delta^{15}N$ values (**fig. S14**). The original values can be accessed online (*49*). The SB dog displays relatively restricted nitrogen and carbon stable isotopes values across all hair and bone samples, averaging 16.3‰ ± 0.5 (mean ± SD) $\delta^{15}N$ and -13.9‰ ± 0.5 $\delta^{13}C$. The hair and bone samples from Mutton have lower $\delta^{15}N$ values than the SB dog, as well as lower $\delta^{13}C$ values, at an average of 10.6‰ ± 0.5 $\delta^{15}N$ and -17.0‰ ± 1.4 $\delta^{13}C$, respectively. The $\delta^{13}C$ values obtained from Mutton's hair and bone samples also have greater variability, ranging from -18.7 to -15.3‰ compared to SB Dog's -14.5 to -13.2‰. Though sample sizes are small, one-way MANOVA shows significant differences between SB Dog and Mutton's stable isotope values, Pillais' Trace = 0.98, $F(2, 10) = 207.59$, $p < 0.001$ (**fig. S13**).

Several $\delta^{13}C$ and $\delta^{15}N$ data from archaeological dogs within the Pacific Northwest have previously been published (mid-Holocene), along with late-Holocene deer from the broad region, which are included here as a herbivore reference (**fig. S14A**) (*22*). While the $\delta^{13}C$ and $\delta^{15}N$ values of the SB Dog align with other archaeological dogs from the broad coastal region, Mutton's bone collagen $\delta^{13}C$ and $\delta^{15}N$ values are notably distinct from coastal dogs, displaying a lower $\delta^{15}N$ value and a lower $\delta^{13}C$. As Mutton lived alongside George Gibbs during the boundary survey of the Canadian-US Border (49th parallel), he and Mutton spent the bulk of their time away from the coast in the elevated mountainous terrain of the Cascade mountains and the Columbia Plateau. This appears to have influenced Mutton's isotopic signature both for bone collagen and hair, as these are consistent with a diet largely lacking in marine foods. However, Mutton's $\delta^{15}N$ value collagen appears to be more than a trophic level higher than the terrestrial deer and are plausibly in alignment with existing isotope data from anadromous sockeye and potamodromous kokanee salmon (*Oncorhynchus nerka)* in the Similkameen and Columbia River systems (*155*). Active or recent harvest of these fish is periodically described in Gibbs' journal by Interior Salish peoples. As he was simultaneously engaged in collecting efforts for the Smithsonian, Gibbs additionally describes regular skinning and specimen preparation activities

for a wide range of birds and mammals that would have been actively observed by Mutton and may have been fed entrails.

The SB Dog remained in its original community where its high δ^{13}C and δ^{15}N values indicate a human-provided diet heavy in marine protein (*156–158*). Incremental hair samples provide a relative time sequence of diet shortly before death and show a consistent diet that is very similar to the bone collagen, the latter capturing a lifetime average of isotope values (**fig. S14B**). This pattern suggests the SB Dog received a consistent diet its entire life, which was likely near the coast given the high level of marine input. It also suggests little to no seasonal shift in diet.

Mutton's δ^{13}C and δ^{15}N values indicate a more complicated dietary history reflecting significant travels while in Gibbs' care and a change from the native diet. Generally, Muttons' δ^{13}C and δ^{15}N values are lower than the SB Dog. The sequential hair δ^{13}C values show a clear shift to more negative values later in life, reflecting C3 plant input or animal protein that consumed more C3 plants (**fig. S14C**) (*159*, *160*). This is likely due to Gibbs movement inland (**fig. S15**) and reliance on more terrestrial grain sources for direct consumption or foddering animals which were fed to Mutton (i.e. pigs, cows, etc.). Historic records of Gibbs' voyage do record a reduced availability of corn and sugarcane (C4), and increased reliance on hunted game such as grouse (more C3-reliant) as their journey progressed (*149*). The sequential hair δ^{15}N values show consistency and similarity to bone collagen values. Without the appropriate baselines and potential dietary source values to conduct a robust isotopic dietary model, we can say only generally that δ^{15}N pattern suggests two potential scenarios for Mutton: 1) a more omnivorous lower protein diet throughout life, or 2) a rapid bone turnover rate during adolescence that captures a significant portion of Mutton's time with Gibbs and the concurrent diet changes as they travelled inland and relied on more terrestrial sources. The first possibility suggests that the SB Dog and Mutton, a woolly dog, would have been fed different diets as part of their native community. The second possibility of rapid bone turnover is perhaps better supported. Mutton's bone collagen values match with the lower range of hair values observed in proximal hair sections which grew just before death, suggesting the bone collagen also reflects relatively recent dietary input. Considering Mutton's young age at death, this scenario is plausible given that bone turnover is more rapid in young mammals of most species.

VIII. Methods: Forensic reconstruction of Mutton

Because Mutton's cranium is not available for study, an archaeological cranial specimen of a male woolly dog was used (**fig. S19A**). The crania, estimated to be ~1,000 years old, was originally excavated from the Little Qualicum River archaeological site on the east coast of Vancouver Island, Qualicum First Nation territory (*4*, *162*). The 3D scan was made at the University of Victoria Library, used with permission from Iain McKechnie, and is hosted on Sketchfab (https://sketchfab.com/3d-models/coast-salish-wool-dog-skull-aa9f839bfdb84347b5da41c8b76e0263). The scan was then simplified and all but the surface geometry was removed, leaving a clean shell (**fig. S19B**). 3D models from scans of canine teeth were downloaded from a collection loaded on Sketchfab by Ludwig-Maximilians University Munich, (**fig. S19C**). The teeth were simplified and sized to fit the skull then mirrored to fit the other side. Because of the wear on the teeth, we believe the dog that the teeth came from was probably quite a bit older than Mutton. The canine teeth were based generally on those of an

Eskimo dog. We then downloaded a dog skull and jaw posted on Sketchfab by Nature Labs, Rhode Island School of Design. The jaw was separated and modified slightly to fit the skull, (**fig. S19D**). Comparing the archaeological woolly dog skull with an American Eskimo Dog skull and a Pomeranian skull, we decided a that Mutton didn't have the toy features of a proportionally shorter maxilla, domed cranium and larger orbits, but had the Mesocephalic skull of a standard American Eskimo Dog or spitz-type dog.

Once the skull, jaw and teeth were assembled, a set of bars representing measurements taken from Mutton's pelt was used as proportion reference for Mutton's spine, ears, tail, and legs (**fig. S19E**). The pelt is distorted by age and the preservation process, so the measurements were not exact representations. More weight was given to the measurements for the metatarsals, ears, and the spine, which likely matched Mutton's proportions in life most closely. A dog without fur was modeled based on the skull, proportions, historical photographs of woolly dogs and written descriptions of Mutton **fig. S19E**).

After review and recommended modifications of the initial hairless model, hair was added to the Mutton reconstruction using Blender 3.5's (https://www.blender.org/download/releases/3-5/) hair curve system and hair particle system. Additional small components were created using Zbrush 2022 fibermesh system (https://www.maxon.net/en/zbrush).
Several haired versions of Mutton were reviewed for color, proportions and an appropriate sense of cleanliness and grooming. The final version of Mutton was rendered in Blender 3.5 and displayed alongside several modern spitz-type breeds (American Eskimo Dog, Alaskan Malamute, Samoyed, Alaskan Husky, Finnish Spitz) besides him for size comparison, (**Fig. 1C**).

IX. Methods: Ethnographic Interviews

Pre-interview preparation
With any project involving Indigenous People it is important to work with community knowledge holders in a respectful manner and with good heart. Before commencing the project, for both the ethnographic component and the scientific analysis, it was decided to first consult with a representative of the Coast Salish Nations who, in the past, had kept and used the dogs for their wool. The woolly dog specimen at the Smithsonian NMNH known as "Mutton" was acquired in 1858-59 near modern day Chilliwack, BC. Although Mutton could have come from elsewhere, it was decided to first discuss the project with the Honorable Steven Point OBC, Chancellor of University of British Columbia, former Lieutenant Governor of British Columbia and Grand Chief of the Stó꞉lō Nation, and his wife Dr. Gwen Point, Chancellor of the University of the Fraser Valley. The Stó꞉lō Nation is a political amalgamation of eleven Stó꞉lō communities whose collective territories extend westward along the Fraser River from the southern point of the Fraser Canyon (Hope) and along the Fraser Valley as far as Langley and including Chilliwack.

With Honorable Steven Point's and Dr. Gwen Point's approval to proceed with our research, an advisory committee was then formed. Coast Salish communities are numerous and cover a large geographic area both north and south of the USA/Canada border, with territories extending

approximately 150 miles both east to west and north to south. This was too large of an area, with too many communities to create an advisory committee with representatives from each community, so a “community of interest” committee was created consisting of Coast Salish people, living both north and south of the border, having publicly expressed an interest in the Coast Salish Woolly dog: Coast Salish weavers, Knowledge Keepers, Elders, and young people. Interview protocols were approved by the Smithsonian Institution Human Subjects Protocol # HS220007, and by the Smithsonian Information and Privacy Office as well as the Research Ethics Board at Vancouver Island University, Nanaimo (#101410), and followed the practice of free and prior informed consent.

The advisory committee helped create, edit, and then review the recruitment documents, the interview agenda, and the questions (around three dozen) for the interviews.

Community interviews and ethnographic documents

We conducted semi-structured interviews focused on woolly dogs in 2022. Interviewees were selected for their knowledge of woolly dogs, their memories, and their concerns on how the history surrounding the dogs has been presented. A total of seven interviews were conducted. Interview questions revolved around understanding the following subjects: the role and value of dogs in society, description of woolly dogs, the use of dog wool in blankets, diet and husbandry of dogs, companionship of dogs, colonial practices/policies that impacted woolly dogs, processing, spinning and weaving the dog wool, and thoughts on how the knowledge gathered from this project should be shared.

All interview recordings, transcriptions and typed field notes were given to the participants for review and approval. The advisory committee also reviewed the summary of the interviews.

Interviewees:

Xweliqwiya Rena Point Bolton, Stó:lō, Elder, 95 years old, Interviewed February 7th, 2022
Danielle Morsette, Master weaver, Suquamish and Shxwhá:y, 34 years old, Interviewed February 4th, 2022
Susan sa'hLa mitSa Pavel, Skokomish, 53 years old, Interviewed February 15th, 2022
Michael Pavel, Skokomish Elder, 63 years old, Interviewed March 4th, 2022
Debra qwasen Sparrow, Master weaver, Musqueam, Interviewed April 15th, 2022
Senaqwila Wyss, Sḵwx̱wú7mesh Úxwumixw (Squamish Nation), Interviewed April 7th, 2022
Eliot Kwulasultun White-Hill, Snuneymuxw, 26 years old, Interviewed June 20th, 2022

Emerging themes

Interviewees’ responses were grouped into themes and key representative quotes for each theme were selected.

<u>The roles of dogs in society</u>

Different roles of dogs were identified, such as wool dogs, hunting dogs, or village dogs, and the dogs were treated differently depending on their role.

> *“You can see that there's different uses, different breeds, different types of jobs and roles that the dogs were in in the community.”*
>
> (Senaqwila Wyss, Sḵwx̱wú7mesh Úxwumixw (Squamish Nation))

> *"My grandfather [*Ed Sparrow born in 1898*] told me that every village had wool dogs, that they were like gold because of course, their fibers were mixed with the mountain goat and then rove [made into a roving for spinning] and spun."*
>
> (Debra qwasen Sparrow, Master weaver, Musqueam)

<u>Description of the woolly dog</u>

Interviewees were able to recall who had told them what the woolly dog looked like. Common descriptions included a medium to small dog, white in color, with a curled tail. The curved tail is reflected in the design work on the Skokomish basketry.

> *"Uncle did talk about the dog in that way that it was always an upturned tail."*
>
> (Susan sa'hLa mitSa Pavel, Skokomish)

<u>The use of dog wool in blankets</u>

Some interviewees were able to describe the processing, spinning and use in blankets. In one case, the interviewee's grandfather could recall the names of the women who were making the yarn and the blanket.

> *"And out of it [*an origin story*] they were given the gift of the wool, and they were able to teach the women how to gather the wool, how to process the wool, how to spin the wool, how to weave with the wool, over time."*
>
> (Michael Pavel, Elder, Skokomish
>
> *"I [*Ed Sparrow born in 1898*] watched my grandmother Spahqia, Thelekwutun's wife Selisya all working on these blankets. They were all talking all the time then.' He said when they were done, after he watched them, he said 'they baked that clay that I remember them baking the clay and I remember them, beating it to powder on the table and then mixing it all together'.... I said did you see it? He said 'Yes', he seen it. In the back shed. And all the old ladies were together, my grandmother's Spahqia, Selisya, Thelekwutun's wife, and he named another one in our language, and he said 'they worked together. They were working on [*a*] big piece you know, not like yours. You guys have small pieces. Big pieces.' he said. 'And big balls of wool they'd already worked on.' I said 'Oh, what was the wool made out [*of*] and he said mountain goat, and those dogs that they kept in pens.' he said.*
>
> (Debra qwasen Sparrow, Master weaver, Musqueam)

<u>Diet of woolly dogs</u>

While the main source of food for the dogs was salmon, some communities fed them elk, others whatever humans ate. A common thread behind the food was that the food was chosen to enhance the wool of the dog.

> *"My teacher, Virginia Adams. She had mentioned that they were only fed like salmon and just really like such a good diet to keep their coats nice and fluffy."*
>
> (Danielle Morsette, Master weaver, Suquamish and Shxwhá:y Stó:lō)

<u>Woolly dog husbandry</u>

It was clear that the dogs had to be separated from the regular village dogs to avoid interbreeding. Some communities used islands to keep them apart, others mentioned pens, and others said the wool dogs were kept inside and the village or hunting dogs were not allowed in. It is not known if the dogs were always separated or only when the females were in heat.

> *"We didn't particularly have an island. We had areas that we dedicated to care for [dogs] and we would have in this case, pens or places to keep them separate, so they didn't run away and we could keep them protected."* (Michael Pavel, Elder, Skokomish)

Woolly dog ownership

A couple of interviewees mentioned that only high-status people kept dogs as it took resources to keep them.

> *"Because only the wealthy women of status had them and they weren't allowed to breed them unless you got permission. Th'etsimiya probably had 10–15 dogs, little dogs."*
>
> (Xweliqwiya Rena Point Bolton, Elder, Stó:lō, 95 years old)

> *There's a village site in Snuneymuxw along the river right near the Cedar Bridge, that translates to two wolves, and it's a story of two supernatural wolves who lived there and walked along the far side of the river. It just seems to me like it's very distinct. With my understanding of our art and the representation of beings in our art, it seems to be a distinct difference between the use of the wool dog or a dog and the wolf, and they carry different meanings within their imagery and the symbolism of it. The wolf would be a helper to people, and so when it's used in their art, that's generally what it means to represent is to honor that connection that they have with the wolf which is a different representation than when people represent the wool dog. I think that the wool dog is more of a representation of wealth for our community to show that we come from high-ranking people. That's kind of an interesting distinction to me when I think about it.*
>
> (Eliot White-Hill, Kwulasultun, Artist, Snuneymuxw)

Colonial policies/practices, cultural genocide, and the demise of the woolly dog

Most accounts of the disappearance of the dog attribute it to the simple explanation of an influx of cheap Hudson Bay Company blankets, but the situation is much more complicated. First, smallpox and other diseases decimated the human populations. The dogs could not survive without caretakers and food. Then colonial policies contributed greatly to the interruption of the culture and the weaving complex.

> *"They were told they couldn't do their cultural things. There was the police, the Indian agent and the priests. The dogs were not allowed. She had to get rid of the dogs. And so the family never ever saw them.* […]
> *No, they were not allowed to keep them because that showed signs of authority and high breeding. The women that had dogs were highborn women and as long as the dogs were there, this reminded the people of who were highborn. And so the dogs were, I don't know what they did to them. They were just either told to get rid of them or they took them… After they took the dogs and what could they do? They only had the mutts that were left around. The ones that they kept for the wool were no longer allowed. And they were the ones that had the long under wool.*
> *We were not allowed, our people were not allowed to spin like on the shxwqáqelets, what do you call it? Yeah, the spindle, yeah spindle whorl. Yeah, they could spin on a European one but not on the shxwqáqelets. And they couldn't*

use their looms, and they would take them out and burn them or they would give them to museums or collectors or whatever, depending on how they were made. I guess you know they were nice to look at and they probably just keep them or sell them, but they confiscated everything and if they caught you making baskets or digging roots or, you know, preparing anything like that, then you would get fined. And if you couldn't pay the fine you went to jail.
So everything came to a halt, everything. The singing, the dancing, the drumming. We were not allowed to have any of those things. The blankets were not allowed and the feather garments that were made for dancing. They were not allowed; they would collect them all and burn them, or they would sell them or whatever, you know. The generation that was there when the Europeans came and colonized us, that's where it ended and there was just a few people who sort of went underground. And my grandmother and my mother were two of them."
(Xweliqwiya Rena Point Bolton, elder, Stó:lō, 95 years old)

"Second, the people who were maintaining the dogs also confronted the racism and discrimination of the non-natives, particularly Indian agents, and they killed the dogs. They didn't want the dog producing wool when those would maintain traditional practices that would prevent their 'civilization'".
(Michael Pavel, Elder, Skokomish)

"I don't remember specifically anything about how the Salish wool dog went extinct in Snuneymuxw, and it's always kind of been really interesting to me too, because I know how significant they were to us and I understand their place within our socio-economic practices.
A lot of what I see from looking online says 'Oh well, it became more convenient to use sheep's wool' so that these dogs just went extinct. Well, that's one way of looking at it, but I don't think that really would have been the case, because these are really cherished dogs to us and that it would have been more convenient or whatever, that doesn't really align with my understanding of our practices and our culture.
I think about when if we're preparing cedar boughs for ceremony, it's really critical that you harvest them before sunrise. You could harvest them anytime around the day, but to us, it's imperative that you do this work in a really specific way, and the protocol is followed. So even if it's less convenient, that's where the energy to it comes from for us.
And so with using the dog wool or the mountain goat wool, as opposed to sheep's wool that could have been purchased in bulk or whatever, I just think that it doesn't really make sense to me, and I think that there's probably more to what was going on, whether it was all of the impacts of colonization and I also think that in this case specifically, with the wool dogs, the impacts of the smallpox epidemic probably can't be understated. Where in many communities only one in 10 people survived, and I can only imagine that it's difficult enough to keep your loved ones alive never mind that the animals that you keep and maintain. That probably had a devastating impact on the wool dog population as well. And then the ongoing erasure and suppression of our culture."
(Eliot White-Hill, Kwulasultun, Artist, Snuneymuxw)

Thoughts on knowledge dissemination of the research

While many interviewees mentioned a publication, there was agreement that audio and video or a documentary would be useful and sharing with communities through presentations or via Zoom.

> *"Part of me says an informational book or little videos like sharing the videos that we had and that kind of stuff. Part of me is like I want it to be detailed records, but it's also how do you make it accessible and palatable for people?"*
>
> (Senaqwila Wyss, Sḵwx̱wú7mesh Úxwumixw (Squamish Nation))

> *"Part of the whole narrative around the woolly dog that I find really interesting is that it starts to unravel, in a way, people's understanding of us as a hunter gatherer society, and that our society was so much more complex than what people took, take it for in general. Hunter gatherer is kind of this dominating narrative that just blankets everything and takes away the complexity and the nuances and our relationship with the woolly dogs clearly shows that there is more complexity to this, and that our relationship with the camas patches and the clam beds and the way that we tended the land and tended the forests, these all show the systems that were in place that are far more complex than what people take for granted about Coast Salish culture. And so it's so much about combating this simplistic aspect or the simplistic lens with which our culture is looked at and showing that actually things are much more complex than many may think."*
>
> (Eliot White-Hill, Kwulasultun, Artist, Snuneymuxw)

> *"Create a publication, so that people can read it, and they can share in that knowledge as well. And maybe it needs to be presented. But it also needs to be given back to the community. And how should that be given back to the community? Well-printed matter, videotapes, audiotapes, or in-person. That's how it should be presented. Let's have a dinner where the people can stand up and provide their own oratory about what all this means, let them express themselves. Let that be an ongoing record. But the thing that's most important out of this is to realize that that wool dog created a gift to produce and to make something to create something to bring something alive. Let's do that. Let's bring that back to life. We want to make sure to realize that the wool dog is still very much a part of our life. And it's generating a conversation, and interaction and outcomes that is the embodiment of goodness."*
>
> (Michael Pavel, Elder, Skokomish)

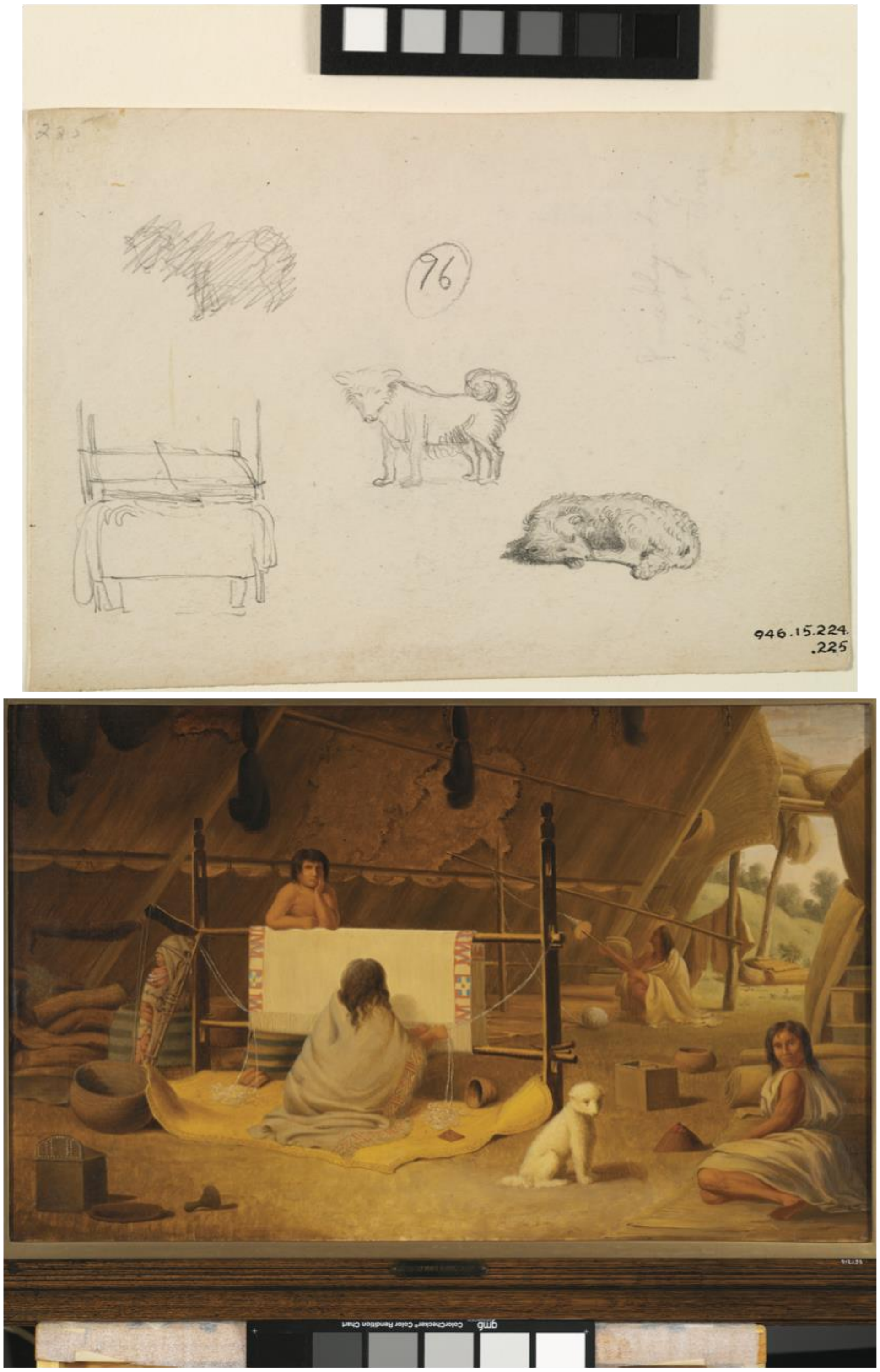

Fig. S1. Sketches and painting featuring woolly dogs by Paul Kane (1849-1856). *Top panel: "Studies of Wool Dogs and Interior Furnishing" (946.15.225), April-June 1847. Bottom panel: "A Woman Weaving a Blanket" (912.1.93), 1949-1856. Courtesy of ROM (Royal Ontario Museum), Toronto, Canada. ©ROM*

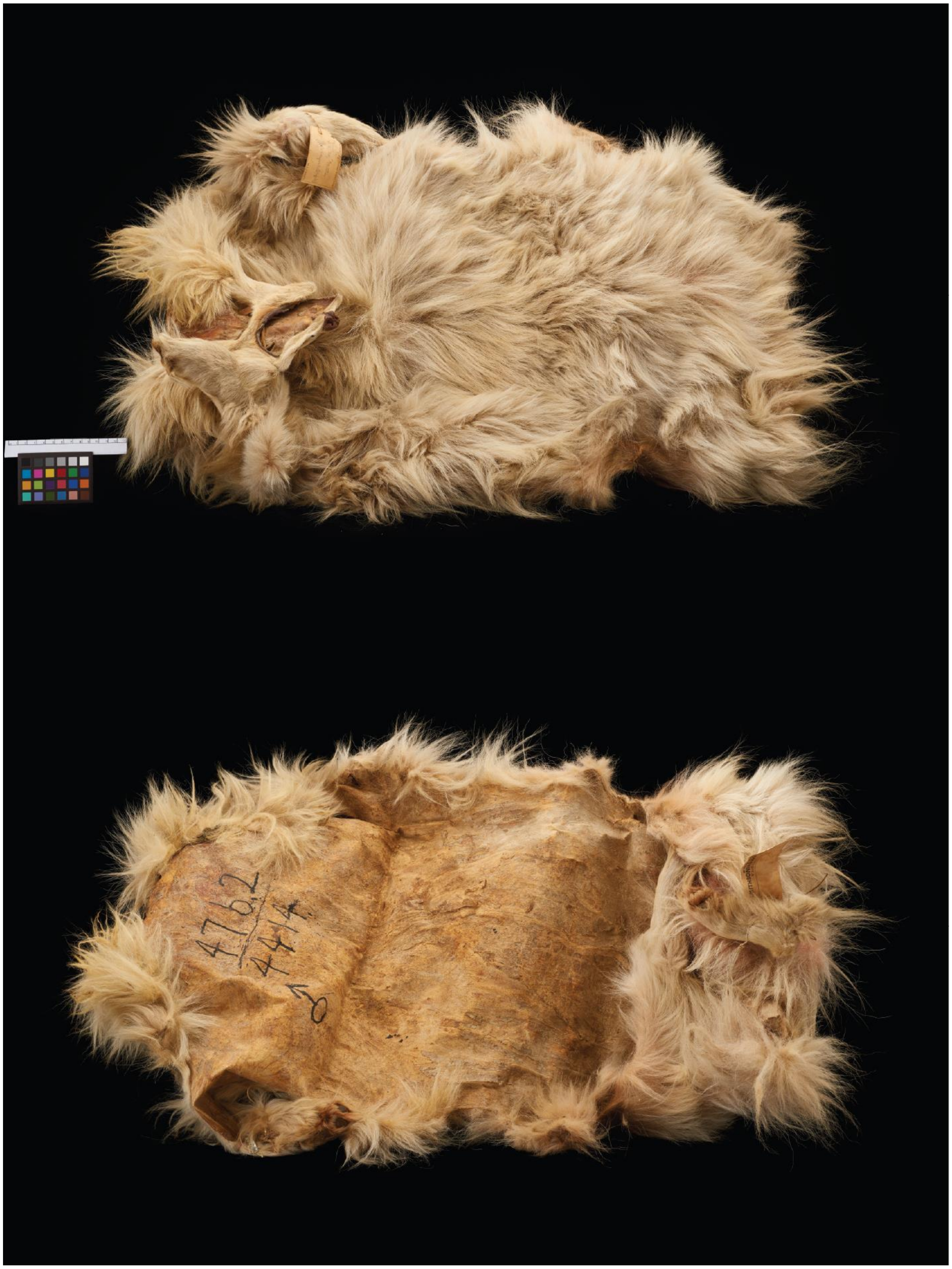

Fig. S2. Mutton Pelt (USNM 4762). *Photograph.*

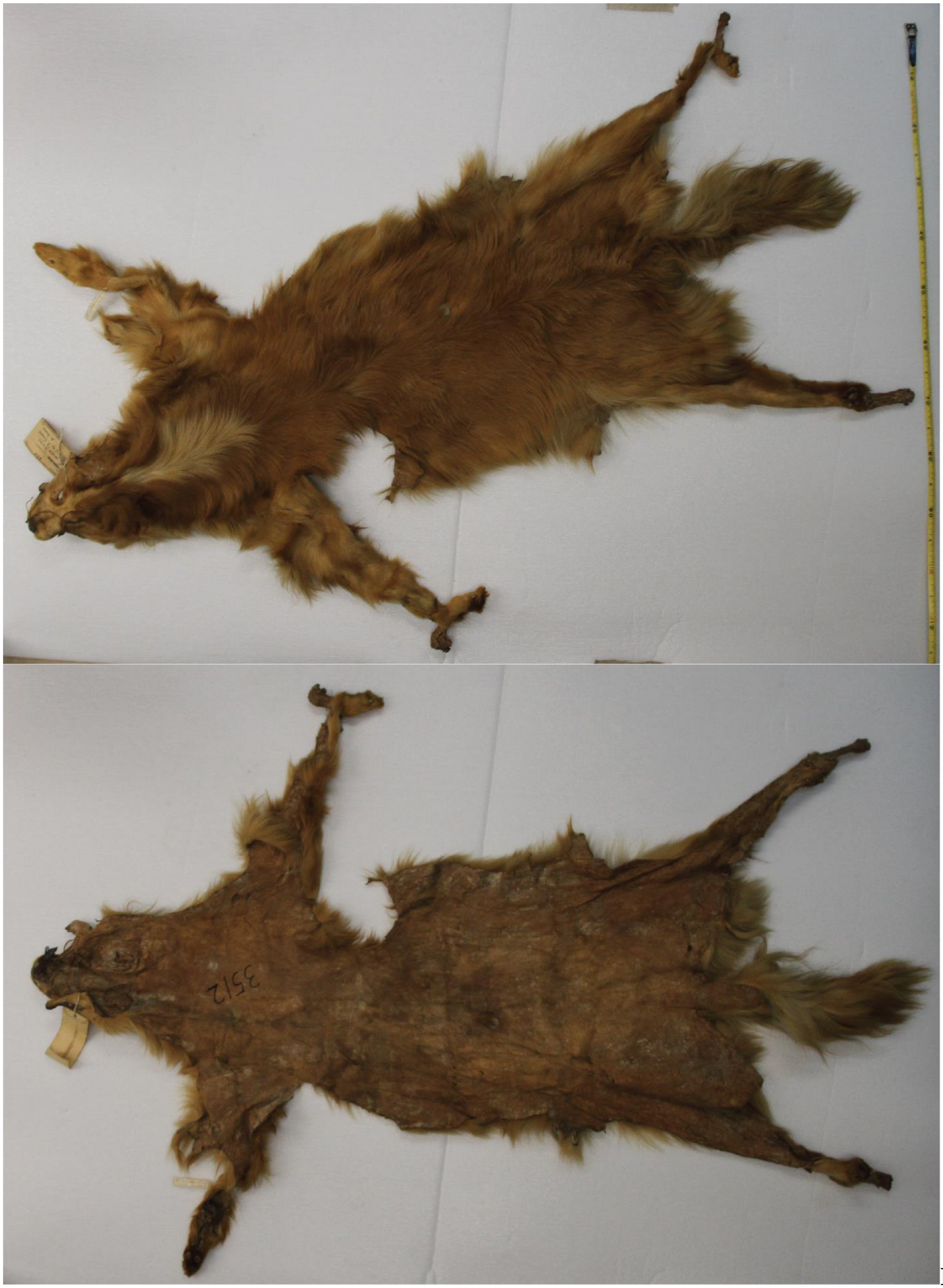

Fig. S3. SB Dog Pelt (USNM 3512). *Photograph.*

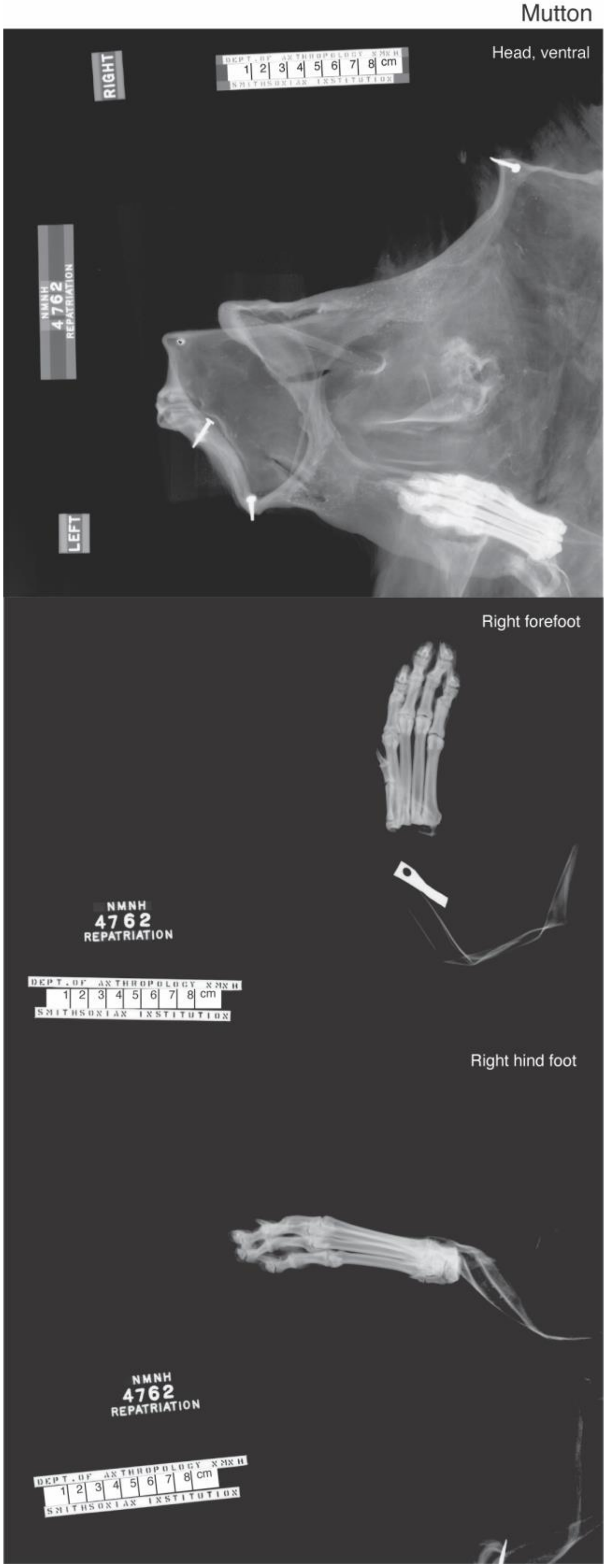

Fig. S4. X-ray of Mutton. *Top - head, ventral. Embedded nails and the left forefoot are visible; middle - right forefoot (front paw) with ID tag, in dorsal position; bottom - right hind foot.*

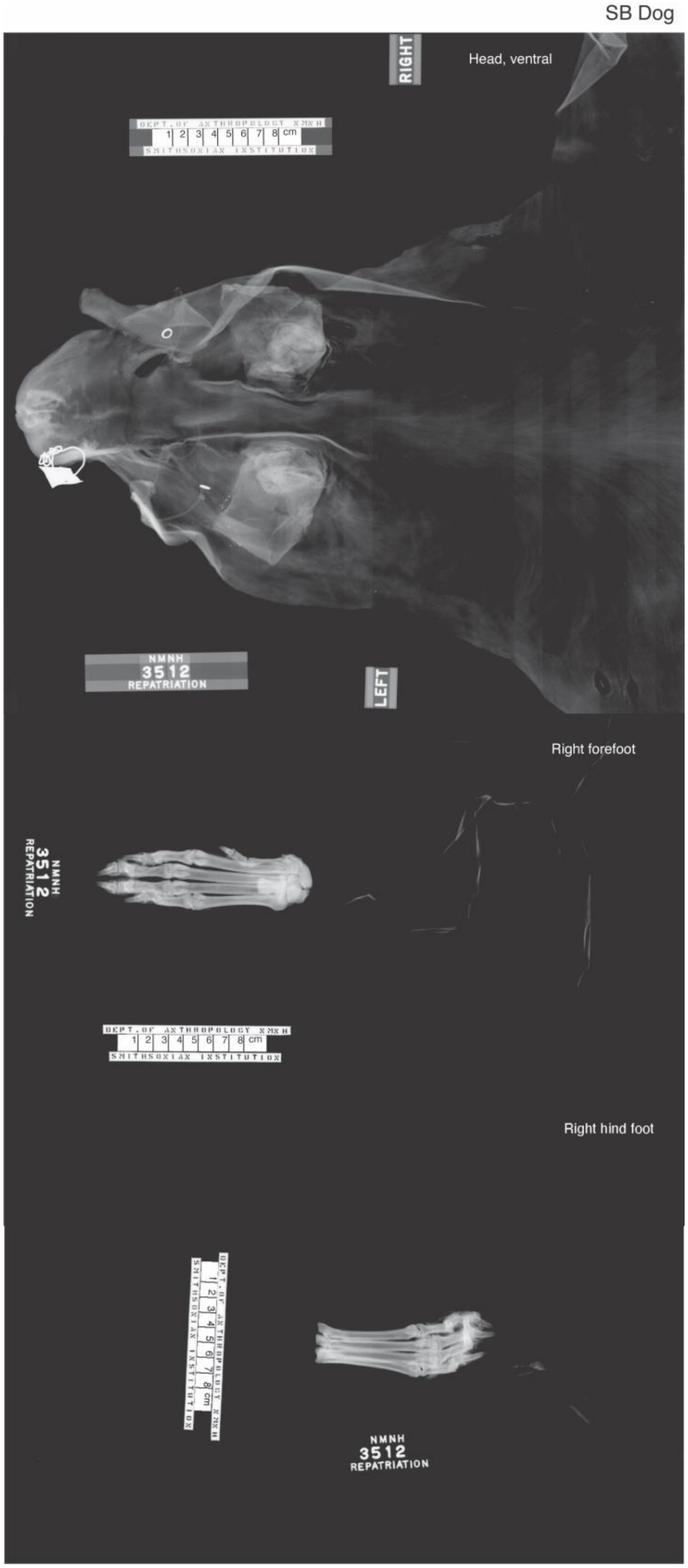

Fig. S5. X-ray of SB Dog. *Top – head, ventral. ID tag is visible.; middle – right forefoot (front paw) in dorsal position; bottom – right hind foot.*

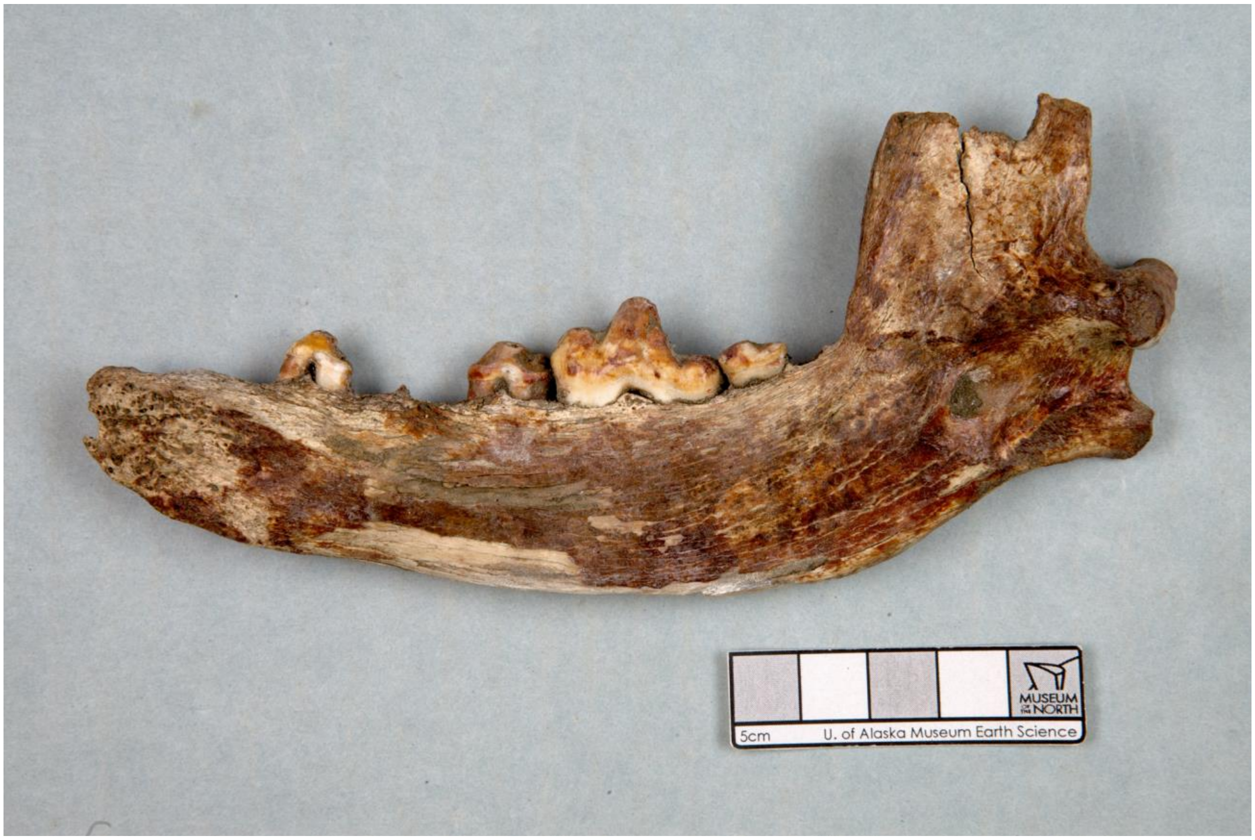

Fig. S6. ALAS_015 dog. *Photo courtesy of University of Alaska Museum of the North.*

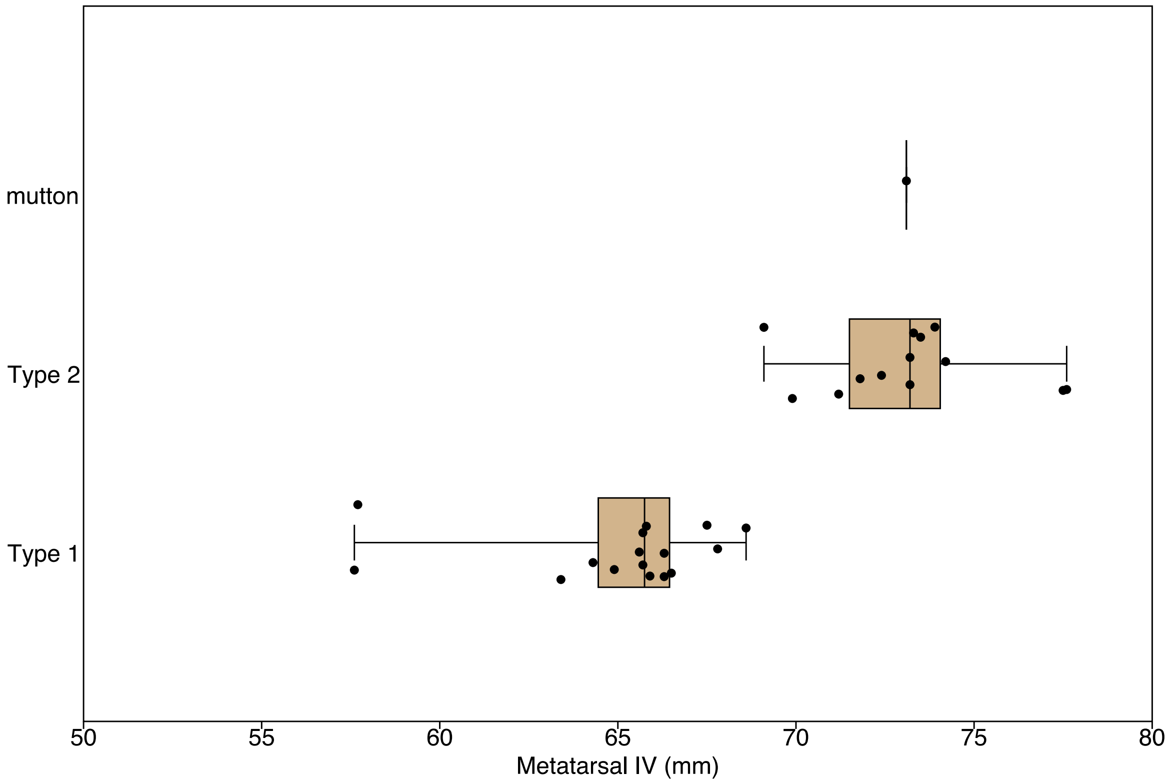

Fig. S7. Metatarsal IV Measurements of Mutton and archaeological dogs. *Archaeological dog data according to Crockford* (94)*: Type 1 "woolly" dogs (*n=*16) & Type 2 "Village" dogs* (n=*13).*

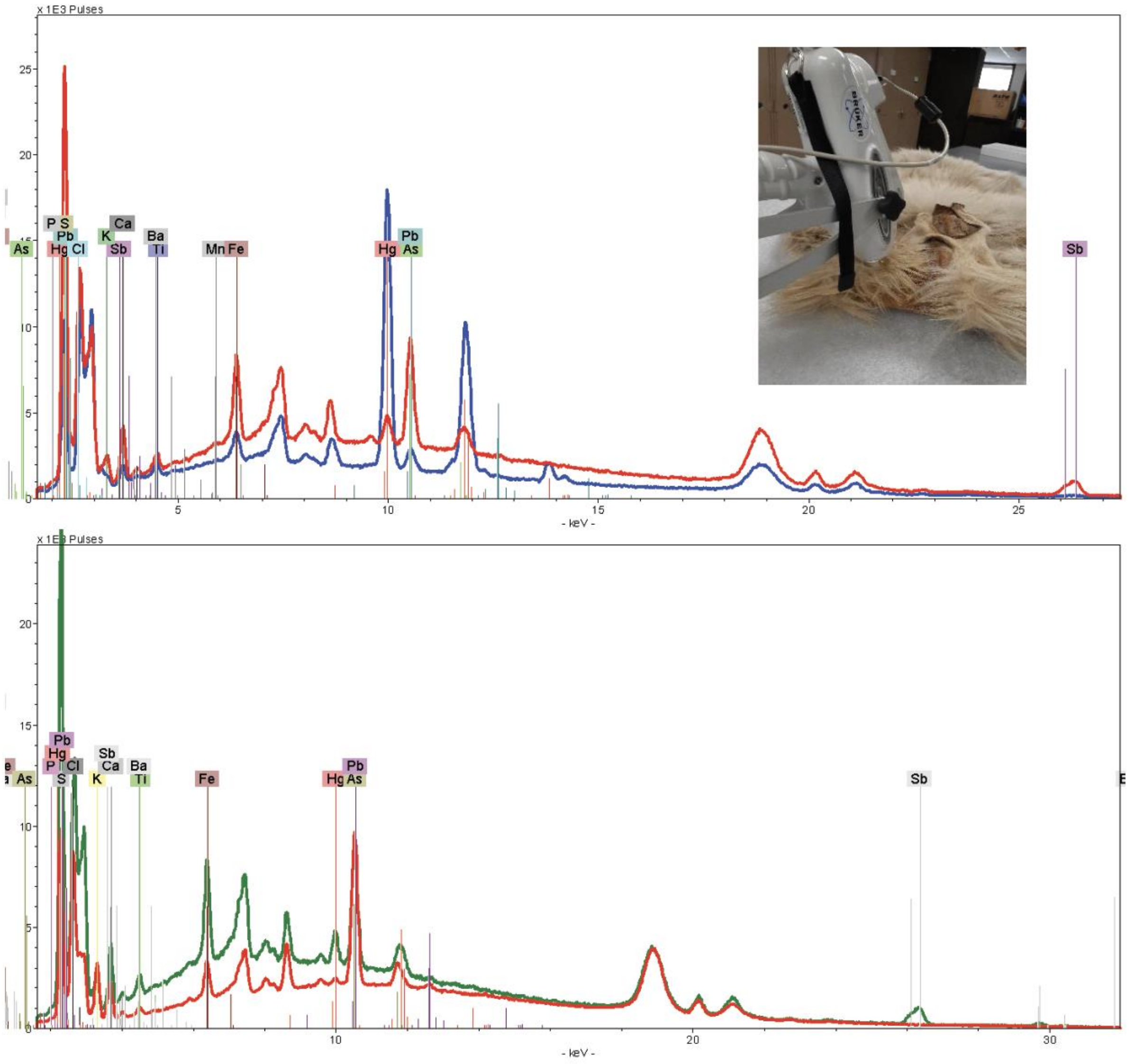

Fig. S8. Overlay of XRF spectra of a pelt from Mutton (USNM 4762) and SB Dog (USNM 3512). *(top) Mutton, Location 01_red hair (blue) and 02_white hair (red). (bottom) Location 02_white hair (green), and SB dog (USNM 3512), location 07 fur head (red).*

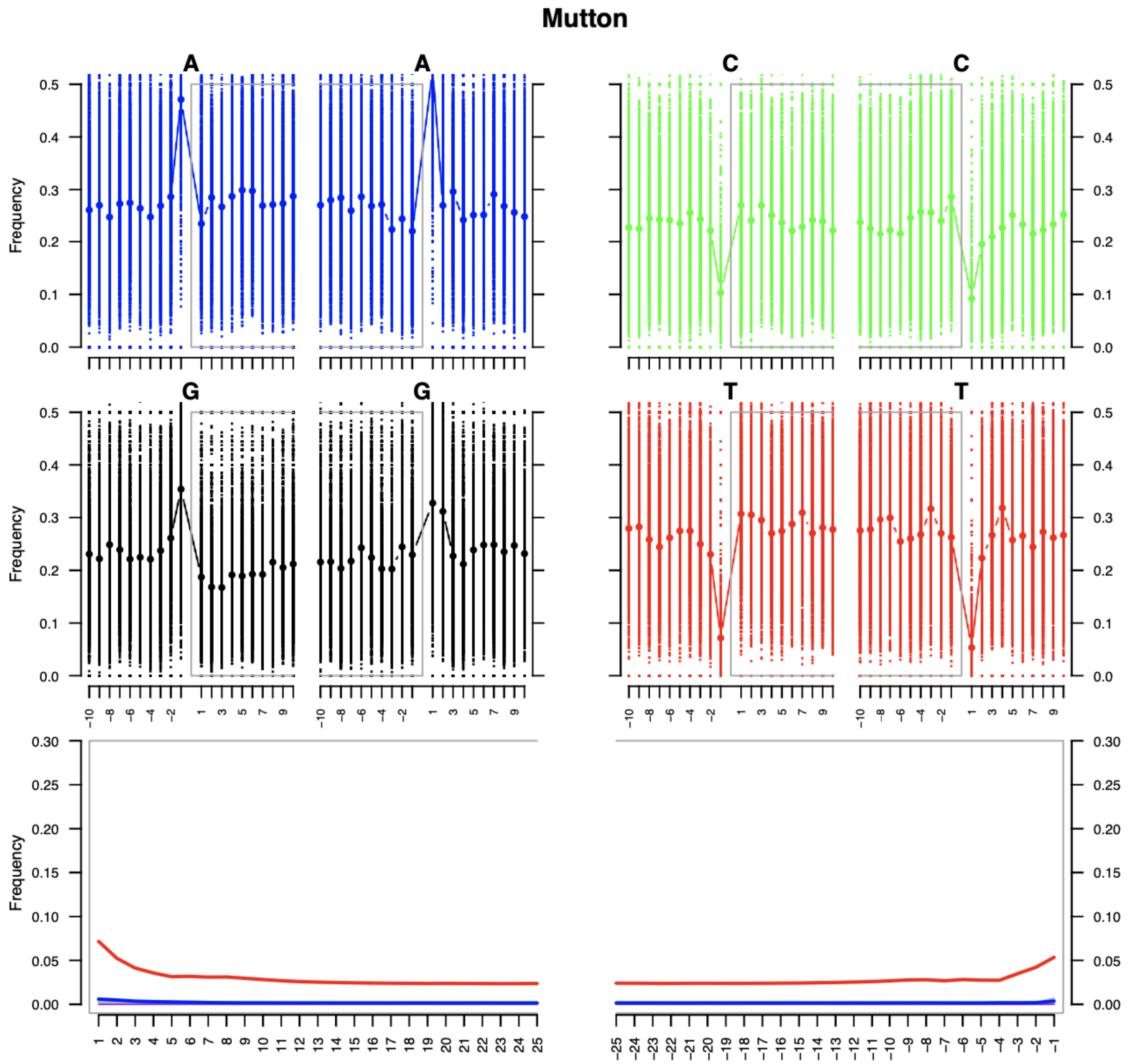

Fig. S9. Mutton MapDamage (*88*) **results**. *Frequency of A, C, G, T nucleotides and the characteristic 5' and 3' damage patterns seen in the bottom panel.*

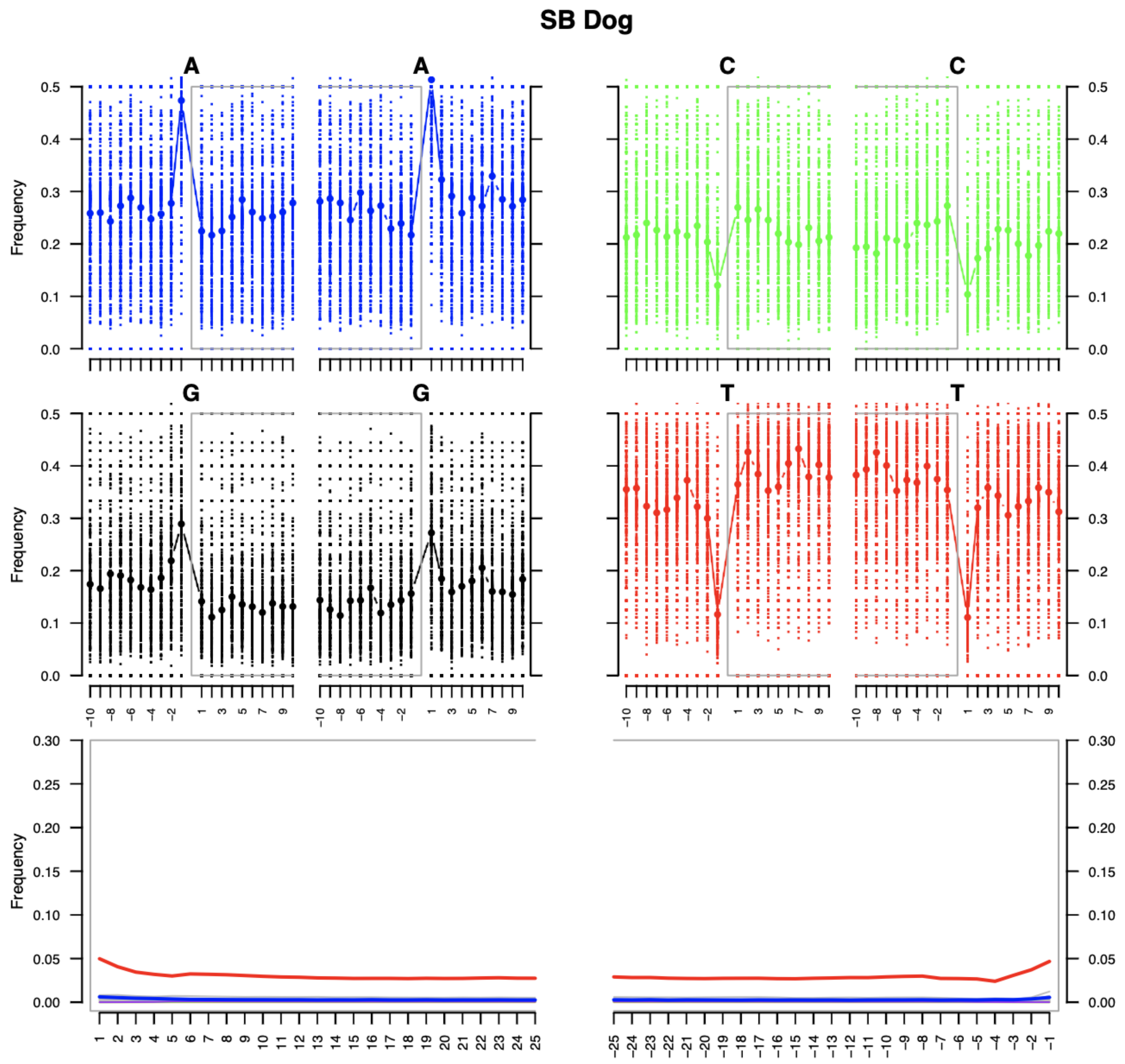

Fig. S10. MapDamage (*88*) **results for SB Dog**. *Frequency of A, C, G, T nucleotides and the characteristic 5' and 3' damage patterns seen in the bottom panel.*

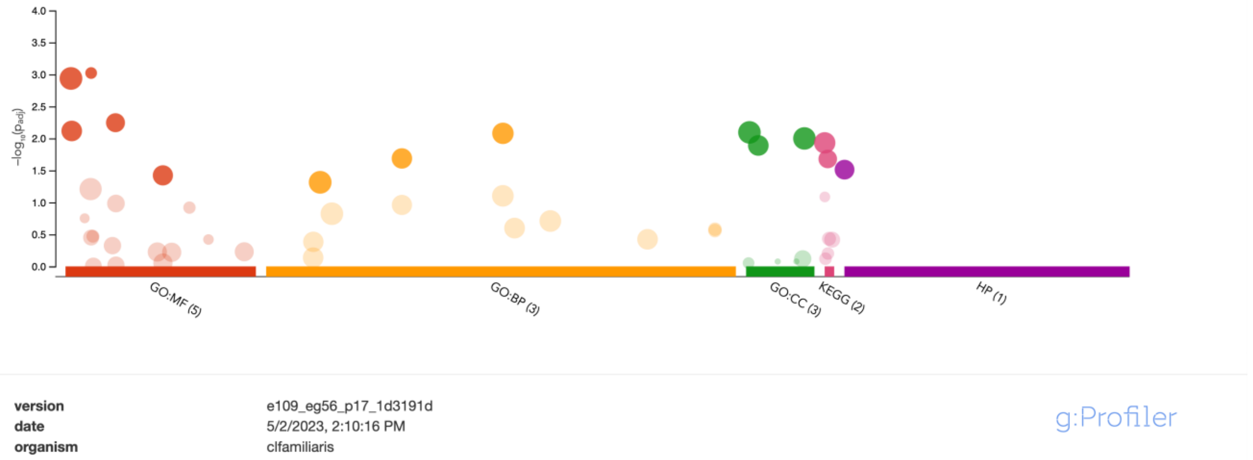

Fig. S11. G:Profiler (*108*) **results after querying 125 enriched genes to the *Canis lupus familiaris* organism**. *Statistical domain scope includes all known genes, g:SCS* (*108*) *significance threshold, and a user threshold of 0.05.*

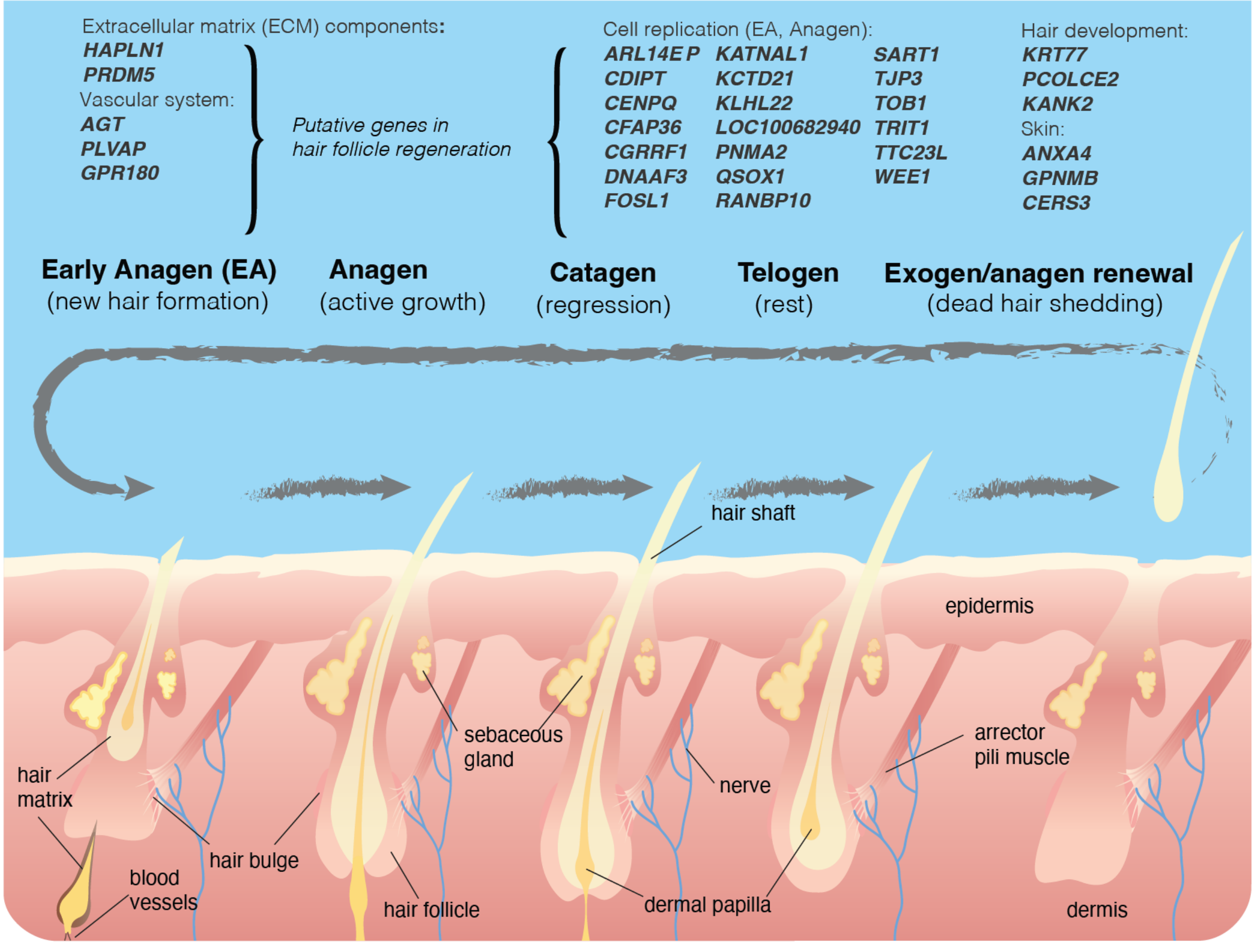

Fig. S12. Proposed model of gene candidates involved in the hair growth cycle of woolly dogs. *The hair growth cycle is complex and involves regulation of stem cell quiescence and activation, cell proliferation, differentiation, and apoptosis* (122). *The hair growth cycle consists of 1) early anagen (EA), where new hair is formed; 2) anagen, the stage of active hair growth; 3) catagen, where the hair stops growing and the hair follicle undergoes apoptosis-driven regression; 4) telogen, the resting phase where the hair follicle is dormant; 5) exogen, where the hair shaft is released. Image modified from the original (created by lembergvector on Freepik).*

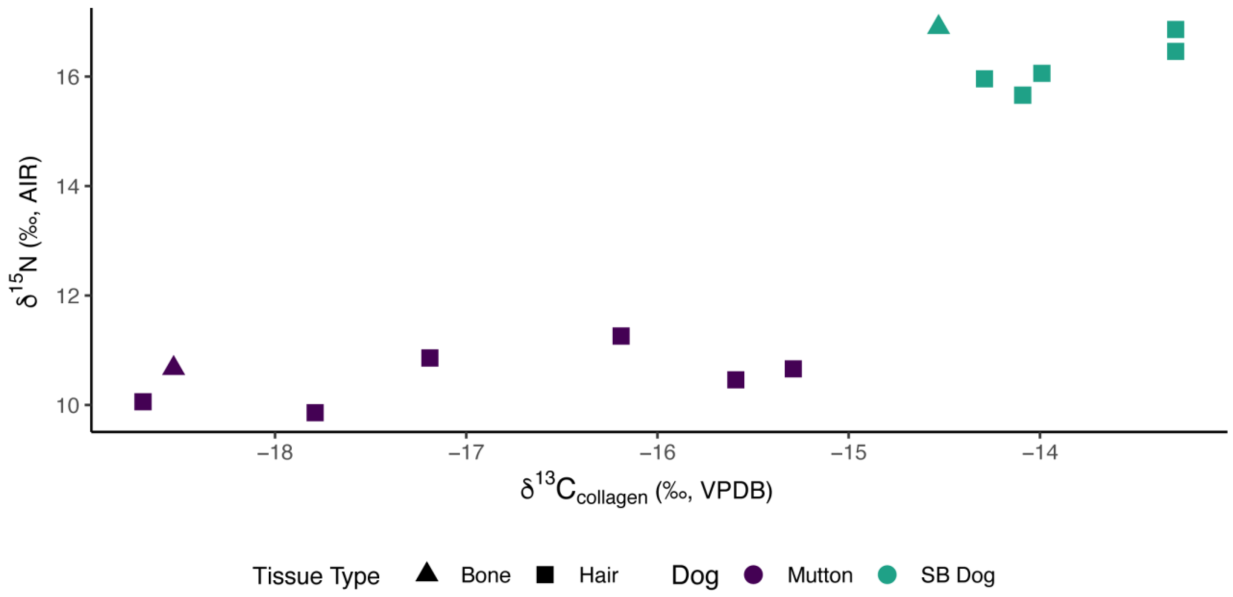

Fig. S13. Stable isotope values of bone collagen and hair keratin. *$\Delta^{13}C$ and $\delta^{15}N$ bone collagen stable isotope values and converted $\delta^{15}N$ hair keratin stable isotope values of Mutton and the SB Dog.*

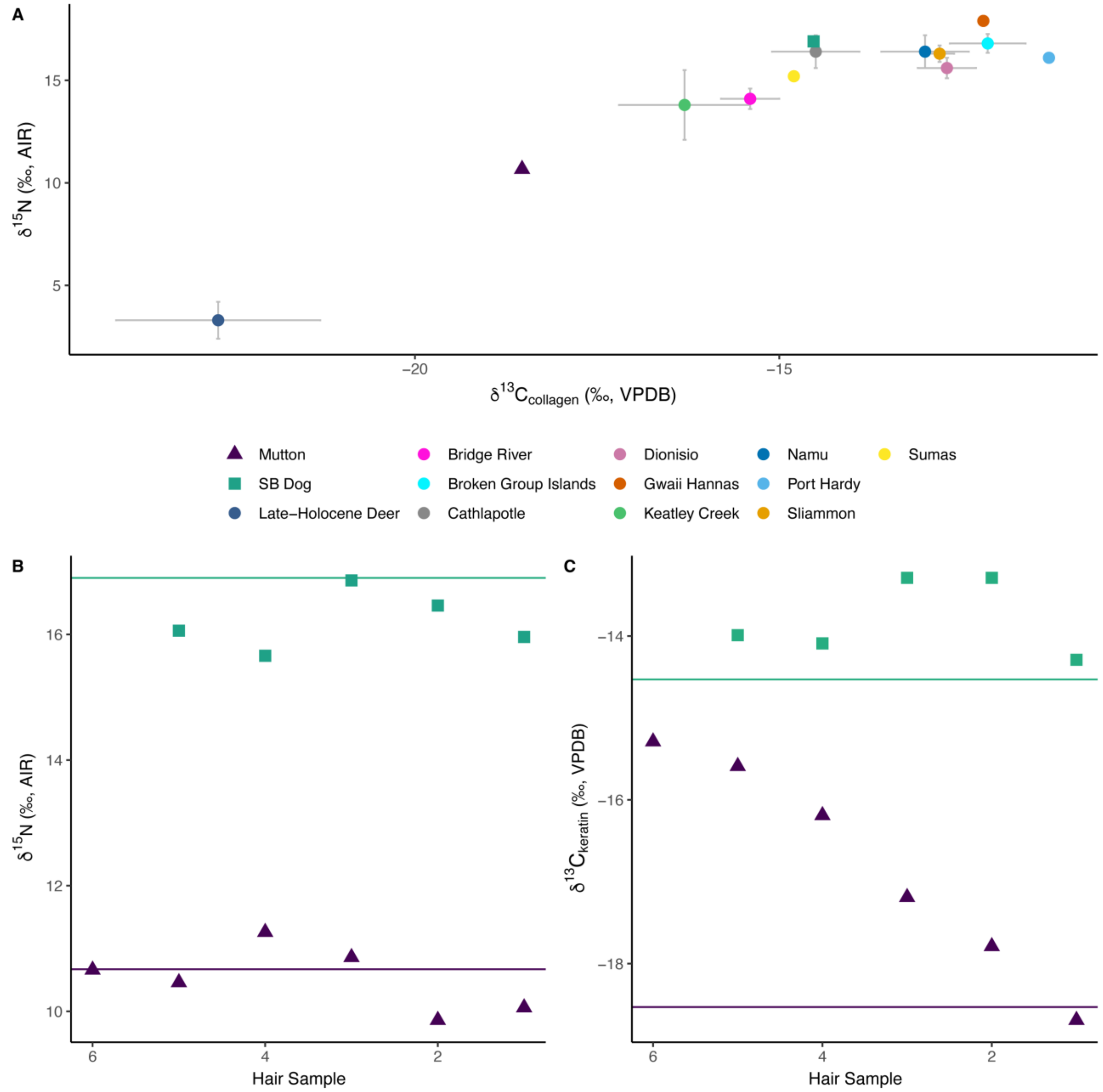

Fig. S14. Stable isotope values of bone collagen and hair keratin. *A) $\delta^{13}C$ and $\delta^{15}N$ bone collagen values of SB Dog and Mutton, plotted with other archaeological dog and deer from* (22)*. B) $\delta^{15}N$ hair keratin values of SB Dog and Mutton (converted to bone values), with bone values as horizontal guidelines. Hair samples are presented with the hair sample representing the oldest time period on the left and the time period right before death on the right. C) $\delta^{13}C$ hair keratin values of SB Dog and Mutton, with bone values as horizontal guidelines. Hair samples are presented with the hair sample representing the oldest time period on the left (e.g. hair 6) and the time period right before death on the right (e.g. hair 1).*

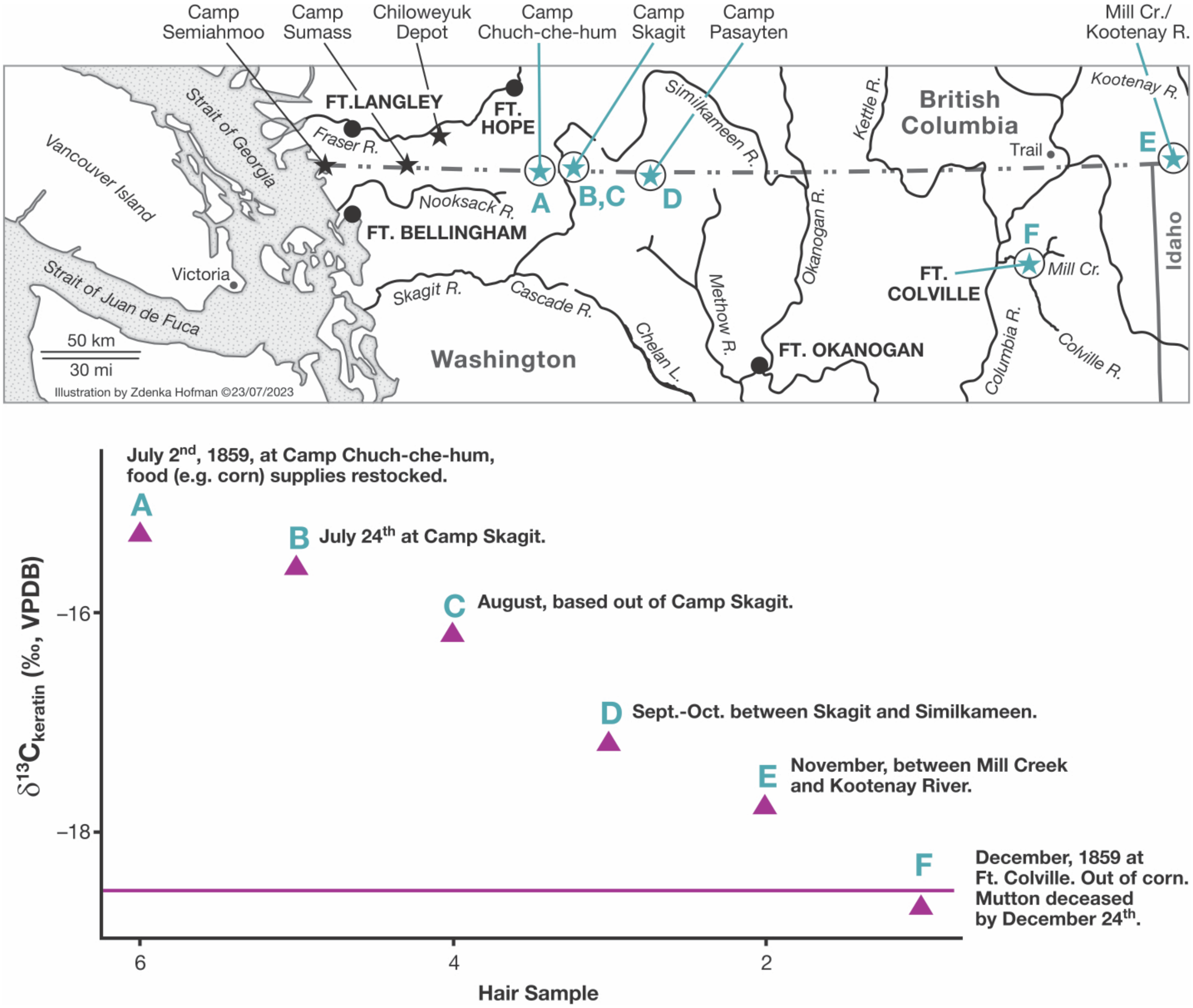

Fig. S15. Sites and stable isotope values of hair keratin corresponding to the last months of Mutton's life. *Chart aligning Mutton's six carbon hair values (triangles) and numbers showing approximate locations where Mutton and George Gibbs may have been for the last few months of Mutton's life. These values are based on the journal entries of Gibbs' and those of the survey team. Supplies (including cornmeal and molasses) were picked up at Camp Chuchchehum and by Ft. Colville corn supplies had run out. Specific dates were from survey team correspondence* (*162*)*, general dates are from Gibbs' field notes* (*15*)*, and geographical coordinates of the camps and stations are from the United States Northwest Boundary Survey* (*65*).

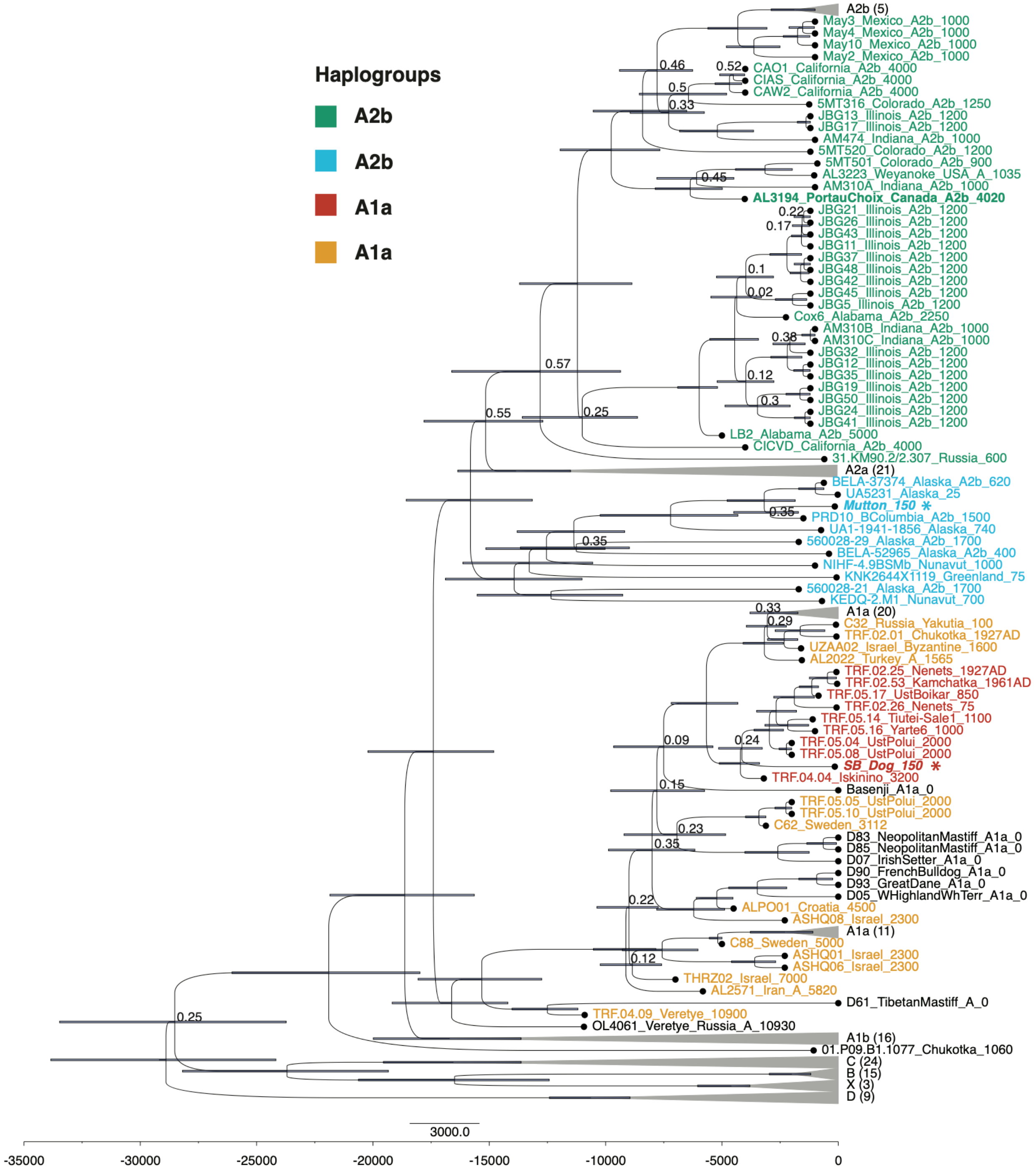

Fig. S16. ***Full mtDNA time tree***. *Colors are of notable dog haplogroups and correspond to* ***Fig 2A****. Black bars at the nodes represent 95% common ancestor highest posterior density. Node posterior support values <60% are labeled. Scale bars indicate years from present.* ***Bolded*** *dogs are newly generated genomes. Mutton and SB Dog are bolded and marked with an asterisk**

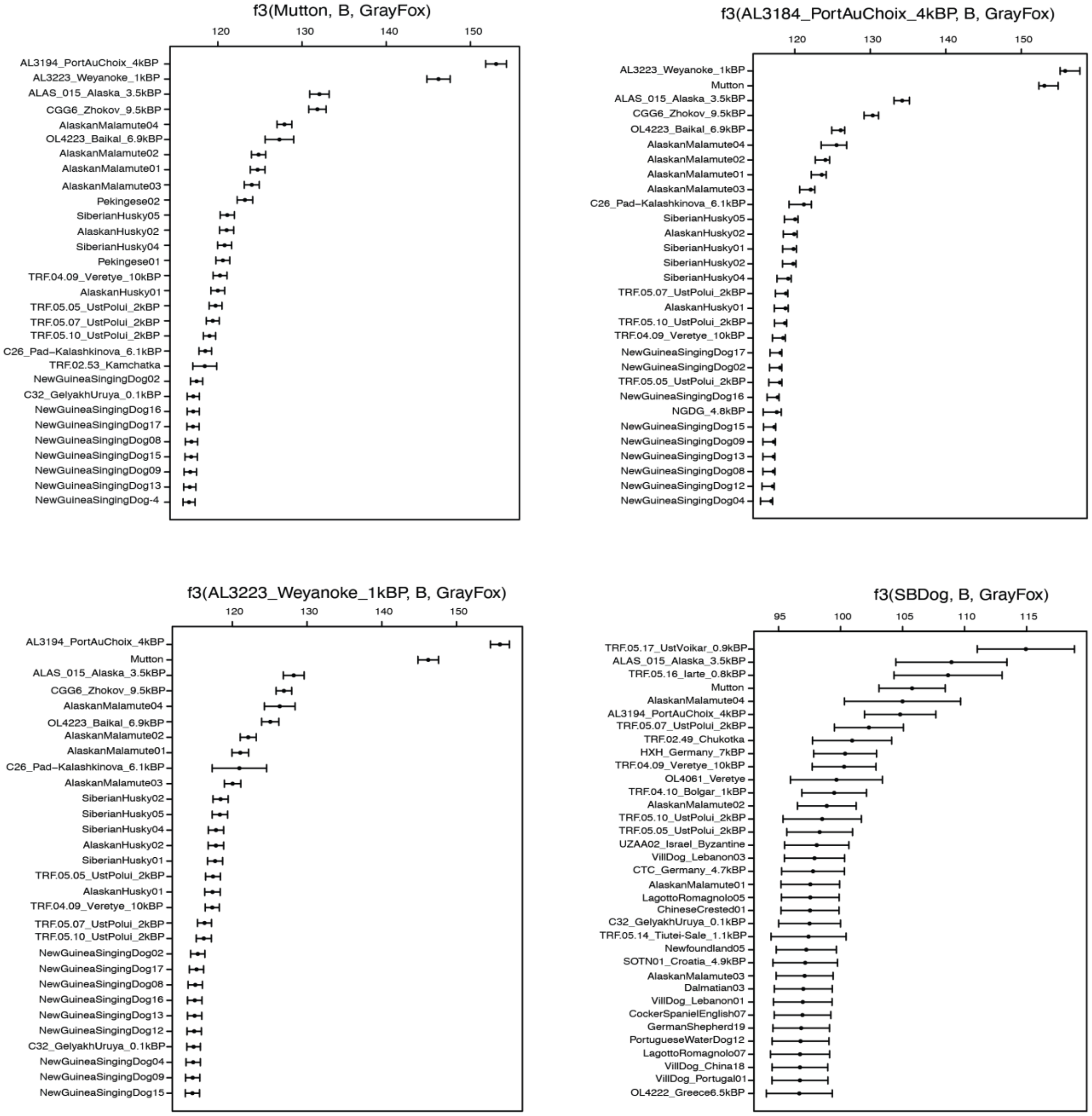

Fig S17. ***F-3 outgroup statistics of Mutton, ancient PCD dogs, and SB Dog***. *Clockwise from top left are plots for Mutton, AL3194 (Port au Choix dog), AL3223 (Weyanoke dog), and SB Dog compared to top 30 ancient and modern dogs, with GrayFox as the outgroup population. Whiskers indicate error bars.*

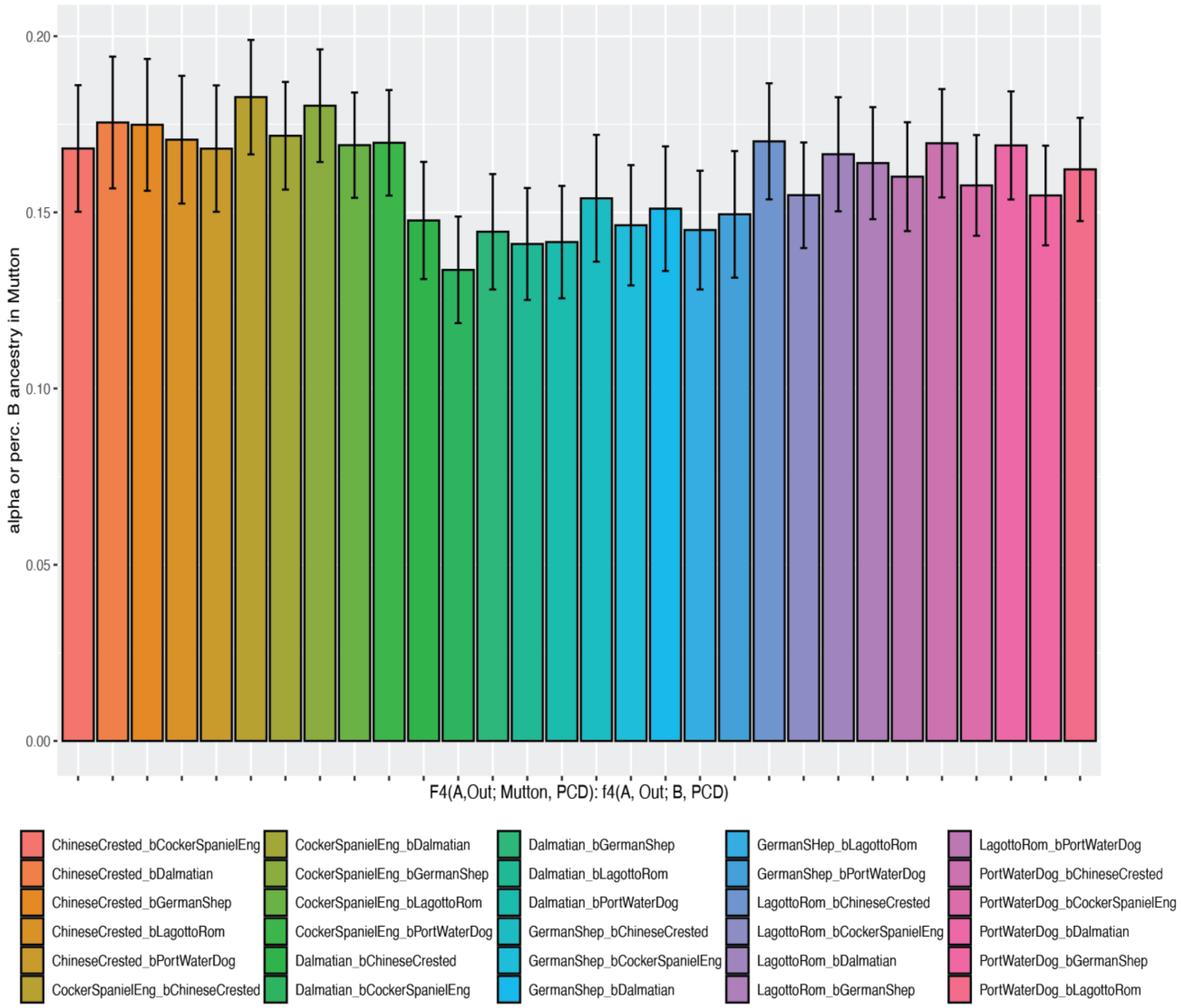

Fig. S18. ***F4-ratio analysis***. *Bar plots of f4-ratio analysis with the following syntax: f4(A, Out; Mutton, AL3194 PortauChoix): f4(A, Out; B, AL3194 PortauChoix) where 6 modern dog breeds (Chinese Crested dog, English Cocker Spaniel, Dalmatian, German Shepherd, Lagotto Romagnolo, and Portuguese Water Dog) are in the A and B placement, and AL3194 (Port au Choix dog) represents a proxy for all PCD dogs.*

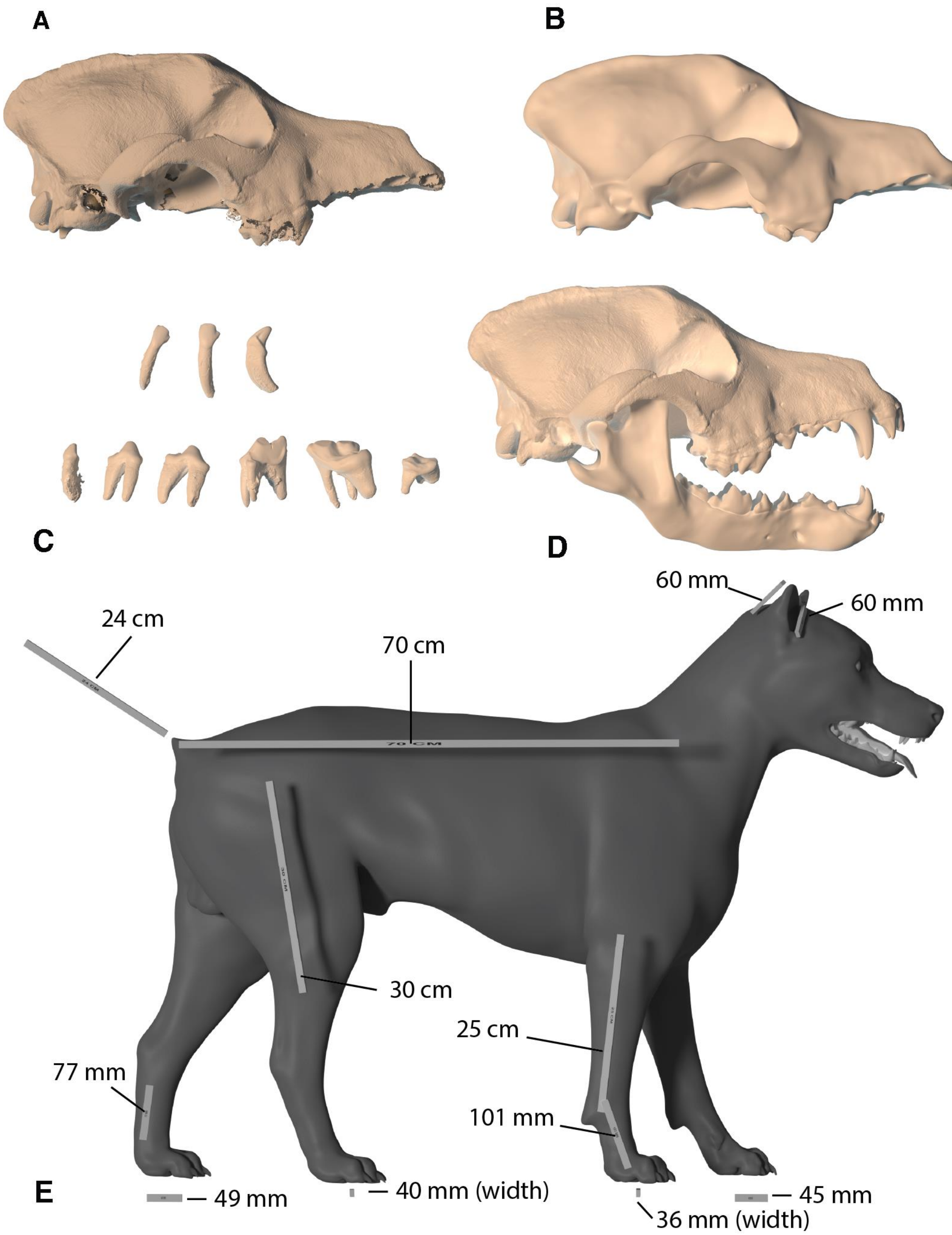

Fig. S19 ***Steps in the forensic reconstruction of Mutton***. *A) 3D model of archaeological woolly dog cranium from Little Qualicum River site* (*4*, *162*) *originally analyzed at the University of Victoria Zooarchaeology lab. The scan was done by UVic library with permission from Iain McKechnie and hosted on Sketchfab (https://sketchfab.com/3d-models/coast-salish-wool-dog-skull-aa9f839bfdb84347b5da41c8b76e0263). B) Simplified and smoothed version of skull scan. C) Teeth fitted to the upper and lower mandibles. Fourth Molar is based on "4th cheek tooth (4th premolar) dog (upper jaw)" (https://sketchfab.com/3d-models/4th-cheek-tooth-4th-premolar-*

dog-upper-jaw-e09fee4434c840a2a6c7a69d70ce70cb) by vetanatMunich (https://sketchfab.com/vetanatMunich) licensed under CC-BY-NC-ND-4.0) (http://creativecommons.org/licenses/by-nc-nd/4.0/). Incisors and molars for are taken from (https://skfb.ly/6TSB6) by vetanatMunich is licensed under CC Attribution-NonCommercial-NoDerivs (http://creativecommons.org/licenses/by-nc-nd/4.0/). D) Teeth fitted to mandible, which was taken from (https://skfb.ly/o67JH) by RISD Nature Lab is licensed under Creative Commons Attribution (http://creativecommons.org/licenses/by/4.0/). E) Hairless model of Mutton with superimposed measurements taken from Mutton's pelt.

Table 1: XRF analysis results of pelt of SB Dog (USNM 3512).

Spectrum Name and Description	Elements Detected	Materials Inferred
6575.10.16_3512_40kv_30uA_01_skin	Major: As<br>Minor: Cl, Ca, Fe, K<br>Trace: S, Ba, Si, P, Sr, Hg, Pb, Mn	Most of the elements detected may be associated to previous preservation treatment.<br>Traces of elements such as Ca, Cl, Fe, K, S, and P may be associated with the skin.
6575.10.16_3512_40kv_30uA_02_skin	Major: As, K, Cl<br>Minor: Ca, Fe<br>Trace: S, Ba, Si, P, Sr, Hg, Pb	Less arsenic and calcium, and more potassium than location 01.
6575.10.16_3512_40kv_30uA_03_bone_backleft	Major: Ca<br>Minor: K, Fe, As<br>Trace: S, Ba, Hg, Sr, P	High amounts of calcium (Ca) consistent with presence of bone.
6575.10.16_3512_40kv_30uA_04_paw_p.r._front	Major: As<br>Minor: Cl, Ca, Fe, K<br>Trace: S, Ba, Si, P, Sr, Hg, Pb, Mn	Similar to location 01 (skin) but less calcium (Ca), and arsenic (As).
6575.10.16_3512_40kv_30uA_05_tag	Major: Cu<br>Minor: -<br>Trace: As, Hg, Cl, Ca, Ba, Pb	High amounts of copper (Cu) associated to the tag. Other trace elements most likely due to previous preservation treatments.
6575.10.16_3512_40kv_30uA_06_papertag	Major: Cu, Zn<br>Minor: -<br>Trace: As, Hg, Cl, Ca, Ba, K	Copper (Cu) and zinc (Zn) detected on paper tag, most likely from the small brass ring. Other trace elements most likely due to contamination from previous preservation treatments.
6575.10.16_3512_40kv_30uA_07_fur_head	Major: As, S, Cl<br>Minor: Ca, Fe, K, Zn<br>Trace: Ba, P, Hg, Pb, Mn	High presence of sulfur (from the fur) and other similar elements detected from previous preservation treatments.

Note: Whenever hypothesis is offered for possible material identification, this should be confirmed with a complementary technique. Other materials are possible. The instrument cannot detect organic materials and materials containing only elements lighter than aluminum. Also, elements present in very small quantities may escape detection. The argon (Ar) peak from the air can be detected when no vacuum pump is used. The rhodium (Rh) peak is due to the instrument tube (as well as traces of palladium (Pd) and possibly nickel (Ni), copper (Cu), and zinc (Zn)).

Table 2: XRF analysis results of Mutton from NMNH collection (USNM 4762).

Spectrum Name and Description	Elements Detected	Materials Inferred
6575.10.16_4762_40kv_30uA_01_redhair	Major: Hg<br>Minor: As<br>Trace: Fe, Ca, Ba/Ti, K, P, Sb, Pb	Red stain contains high levels of mercury (Hg).
6575.10.16_4762_40kv_30uA_02_whitehair	Major: S<br>Minor: Cl, As, Fe, Sb<br>Trace: Ca, Ba/Ti, K, P, Mn, Hg, Pb	High presence of sulfur (from the fur) and other similar elements detected from previous preservation treatments (such as chlorine, arsenic, and antimony).
6575.10.16_4762_40kv_30uA_03_whitehairfront	Major: S<br>Minor: Cl, As, Fe, K<br>Trace: Ca, Ba/Ti, P, Mn, Hg, Pb	Similar to location 02 but no antimony (Sb) and more potassium (K).
6575.10.16_4762_40kv_30uA_04_redhairfront	Major: Hg<br>Minor: As, K<br>Trace: Fe, Ca, Ba/Ti, P, Pb	Similar to location 01 but slightly more potassium (K).
6575.10.16_4762_40kv_30uA_05_skinfront	Major: K, As, Sb<br>Minor: Cl, S, Fe, P<br>Trace: Ca, Ba/Ti, Mn, Hg, Pb	Highlighting elements used for treating the skin and/or associated with the skin composition. High potassium (K), antimony (Sb), and arsenic (As). Slightly higher content of phosphorus (P).
6575.10.16_4762_40kv_30uA_06_nail	Major: Fe<br>Minor: As, K<br>Trace: S, Cl, Hg, Sb, Ca, Mn, Zn	Iron nail. Notable amount of arsenic (As) and potassium (K).

Note: Whenever hypothesis is offered for possible material identification, this should be confirmed with a complementary technique. Other materials are possible. The instrument cannot detect organic materials and materials containing only elements lighter than aluminum. Also, elements present in very small quantities may escape detection. The argon (Ar) peak from the air can be detected when no vacuum pump is used. The rhodium (Rh) peak is due to the instrument tube (as well as traces of palladium (Pd) and possibly nickel (Ni)). On the spectra, only the elements related to the samples have been labelled.

DataS1. [Supplementary spreadsheet]
IDs and metadata of newly generated genomes (NewGenomesMetadata), Extracts data from Mutton and SB Dog (ExtractsData), estimated error rates in ancient genomes used (AncientGenomeError), samples and metadata for mtDNA analyses (mtDNAdataset), samples and metadata for RoHan analysis (RoHanDataset), samples and metadata for dn/dS analysis (dNdSDataset), samples and metadata for outgroup-*f3* analyses (f3Dataset).

DataS2. [Supplementary spreadsheet]
g:Profiler (*108*) results after querying 125 genes. Separate tabs show results within the categories in GO: Molecular Function (GO_MF), GO: Biological Process (GO_BP), GO: Cellular Component (GO_CC), KEGG, and Human Phenotype Ontology (HP), gene list with dN/dS values in Mutton (mutton_dndList), hypergeometric test results for gene enrichment (res_Hypergeometric), Wilcoxon rank-sum test results for gene enrichment (res_RankSum), Gene Ontology Resource query results for several hair/skin genes (AmiGO2).

DataS3. [Supplementary spreadsheet]
125 gene list annotated manually (Annotations) by DAVID (*110*, *111*), (geneList), and results of querying hair and skin categories in MGI Gene Ontology database (https://www.informatics.jax.org/) (MGI_GO_MP_Databases).

DataS4. [Supplementary spreadsheet]
Mutton's genotype of variants associated with hair phenotype in dogs.

Data S5. [Supplementary spreadsheet]
Bone collagen and hair keratin δ13C and δ15N values of Mutton, SB Dog, and referenced comparative dog bone collagen data from previous research in the PNW (*22*).
